# Identification of probe-quality degraders for Poly(ADP-ribose) polymerase-1 (PARP-1)

**DOI:** 10.1080/14756366.2020.1804382

**Published:** 2020-08-11

**Authors:** Zhimin Zhang, Xinyue Chang, Chixiao Zhang, Shenxin Zeng, Meihao Liang, Zhen Ma, Zunyuan Wang, Wenhai Huang, Zhengrong Shen

**Affiliations:** Key Laboratory of Neuropsychiatric Drug Research of Zhejiang Province, Hangzhou Medical College, Hangzhou, P. R. China

**Keywords:** PARP-1, PROTAC, target protein knockdown

## Abstract

Poly(ADP-ribose) polymerase-1 (PARP-1), a critical DNA repair enzyme in the base excision repair pathway, has been pursued as an attractive cancer therapeutic target. Intervention with PARP-1 has been proved to be more sensitive to cancer cells carrying *BRCA1/2* mutations. Several PARP-1 inhibitors have been available on market for the treatment of breast, ovarian and prostatic cancer. Promisingly, the newly developed proteolysis targeting chimaeras (PROTACs) may provide a more potential strategy based on the degradation of PARP-1. Here we report the design, synthesis, and evaluation of a proteolysis targeting chimaera (PROTAC) based on the combination of PARP-1 inhibitor olaparib and the CRBN (cereblon) ligand lenalidomide. In SW620 cells, our probe-quality degrader compound **2** effectively induced PARP-1 degradation which results in anti-proliferation, cells apoptosis, cell cycle arresting, and cancer cells migratory inhibition. Thus, our findings qualify a new chemical probe for PARP-1 knockdown.

## Graphical Abstract

**Figure:**
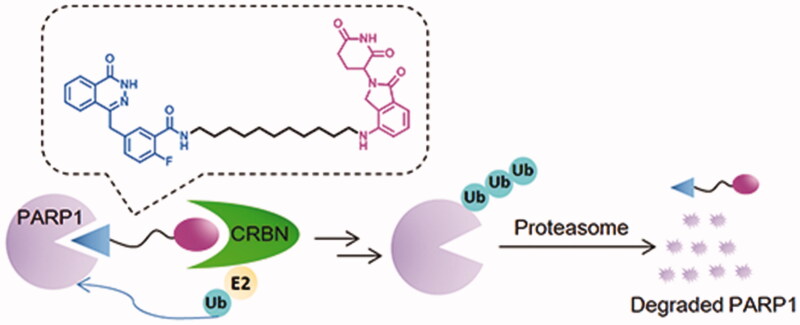

## Introduction

**Figure 1.**
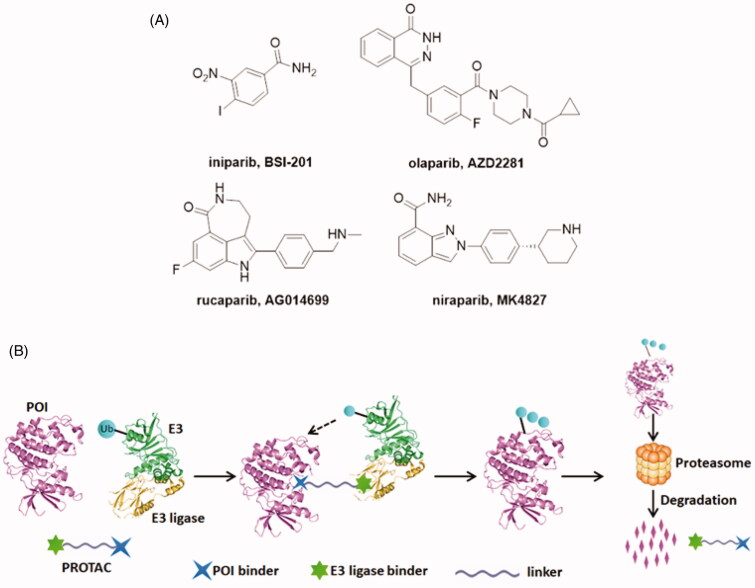
(A) Chemical structures of representative PARP-1 inhibitors. (B) Mechanism of action of PROTAC conjugates (POI: protein of interest; Ub: ubiquitin).

Poly (ADP-ribose) polymerases (PARPs), a family of seventeen protein members, are DNA-dependent nuclear enzymes that participate in DNA damage repair by recognising and rapid binding DNA single-strand breaks (SSBs)^1–3^. Then, SSBs are repaired *via* the base excision repair pathway^4^. Among this family, PARP-1 is the most widely investigated. PARP-1 is generally known to involve in a wide range of cellular functions, such as cell division and differentiation, as well as apoptosis and chromosome stability^5^^,^^6^. PARP-1 knockout animals and cells showed high sensitivity when exposed to γ irradiation and alkylating agents^7^. Elevated PARP-1 expression is always observed in many diseases, such as breast cancer, melanomas, and lung cancer^8^. Due to its pivotal role in DNA damage response, inhibition of PARP-1 is emerging as a useful therapeutic approach for cancers^9–11^. Until now, significant advances and breakthroughs have been achieved in developing PARP-1 inhibitors. Unfortunately, the first PARP-1 inhibitor, niparib (Figure 1(A))^12^, was announced to be unsuccessful when tested in phase III clinical trials in 2011^13^. The clinical development of niparib was not going smoothly but was ultimately successful and other three PARP-1 inhibitors olaparib^14^, rucaparib^15^, and niraparib^16^ have been approved by the US FDA (Figure 1(A)). The mechanism of PARP-1 inhibitors is synthetic lethality of proteins, which can prevent the DNA repair progress of tumour cells. Some studies have indicated that cancer cells carrying *BRCA1/2* mutations are 1000 times more sensitive to PARP inhibitors than cancer cells carrying wild-type *BRCA*. However, *BRCA1* or *BRCA2* mutations account for only a small percentage of all breast cancers and ovarian cancers. Due to competitive- and occupancy-driven process of PARP-1 inhibitors, their clinical therapies are limited by poor prognosis, complicated heterogeneity and drug resistance^17^^,^^18^.

Recently, targeted protein degradation using Proteolysis Targeting Chimaeras (PROTACs) has emerged as an attractive therapeutic modality in drug discovery^19^. PROTACs are small molecules consisting of three components: a specific ligand to the protein of interest (POI), a moiety specifically recruiting an E3 ligase and a linker that couples these two functionalities^20^. The PROTAC forms a complex upon binding to both its E3 ubiquitin ligase target and the POI and then followed by poly-ubiquitination (Ub) of the POI and its subsequent degradation by the proteasome (Figure 1(B))^21^. At present, four E3 ligases MDM2, clAP1, VHL and CRBN (cereblon) have significantly advanced the PROTAC technology^22^^,^^23^. To date, the PROTAC concept has been widely applied to induce the degradation of various proteins such as kinases, epigenetic reader proteins, nuclear receptors, and transcription factors^24–31^.

An appealing feature for PROTACs is their catalytic, event-driven modality of action, which means that it does not need lasting-binding to target protein in high concentration, so every single molecule could execute multiple rounds of protein degradation. As a consequence, the dosage for treatment can be greatly reduced^21^. Therefore, effective pharmacological degradation of PARP-1 is expected to display minimal toxicity in catalytic amount. In addition, we were highly interested in probing the cellular effects of inhibiting PARP-1 by PROTACs, not by occupancy-based small molecule inhibitors.

In the present study, we proposed to use the PROTAC strategy to develop the probe-quality small molecule degraders targeting PARP-1. Structure-guided conjugation of the FDA approved PARP-1 inhibitor olaparib to a CRBN ligand lenalidomide resulted in the discovery of PARP-1 degraders. We have evaluated the degradation efficacy and anti-proliferative activity of these PROTACs in colorectal adenocarcinoma SW620 cell line. The pharmacological mechanisms, *in vitro* pharmacokinetics of the selected compounds were also presented.

## Results and discussion

### Design of PROTACs target to PARP-1

**Figure 2.**
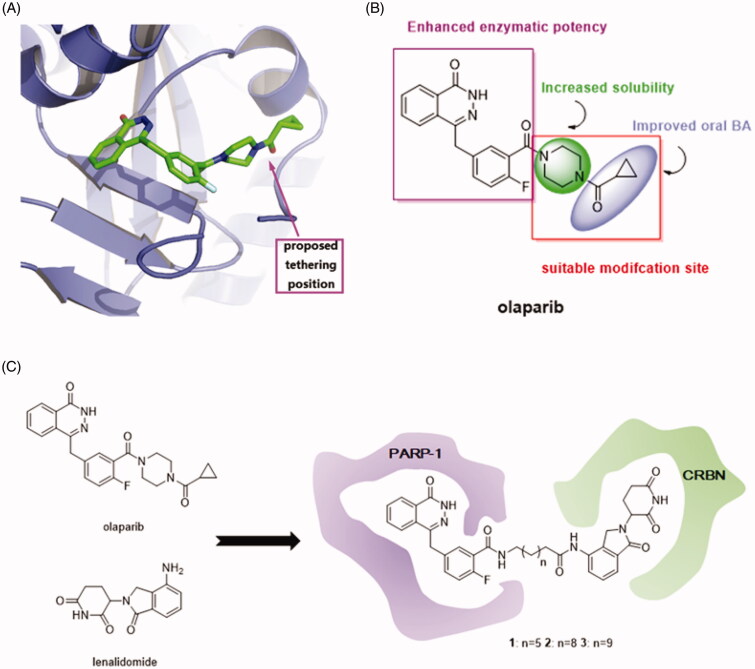
(A) Proposed tethering position on olaparib based upon the co-crystal structure of olaparib in complex with PARP-1 (PDB ID: 4TKG). (B) The SAR of olaparib. (C) Design of PARP-1 degraders based upon the PROTAC concept.

In consideration of the high potency and exquisite selectivity of olaparib, we selected it as the POI moiety. The analysis of the crystal structure of olaparib in complex with PARP-1 indicated that the cyclopropyl(piperazin-1-yl)methanone group of olaparib is solvent exposed (Figure 2(A)). We therefore hypothesised that modification on this site may not losing too much binding affinity. Indeed, the structure-activity relationship (SAR) studies suggested that the diacylpiperazine moiety of this molecule (light-green, Figure 2(B)) improved the solubility and the cyclopropyl group (light-purple, Figure 2(B)) conferred oral bioavailability^14^. Neither of these two parts are crucial for enzymatic potency and substitution of them were well tolerated. Thus, we employed the carbonyl group on olaparib as the tethering site for the design of PARP-1 degraders. On the other way, the design of PROTAC needs an E3 ligase ligand degradation system. Because lenalidomide that binds to CRBN have been widely used for the establishment of PROTACs^32^, we investigated it for the design of PARP-1 degraders. Additionally, lenalidomide was picked over other E3 ubiquitin ligase ligands, such as MDM2, clAP1, and VHL ligands, due to its straightforward synthetic accessibility. The solvent exposed terminal amino group of lenalidomide was conjugated to the alkyl linker *via* an amide bond without perturbing the interaction with the E3 ligases. Accordingly, we employed olaparib and lenalidomide for the design of three initial, putative PARP-1 degraders **1–3** (Figure 2(C)).

### General synthetic route to designed compounds

**Figure 3.**
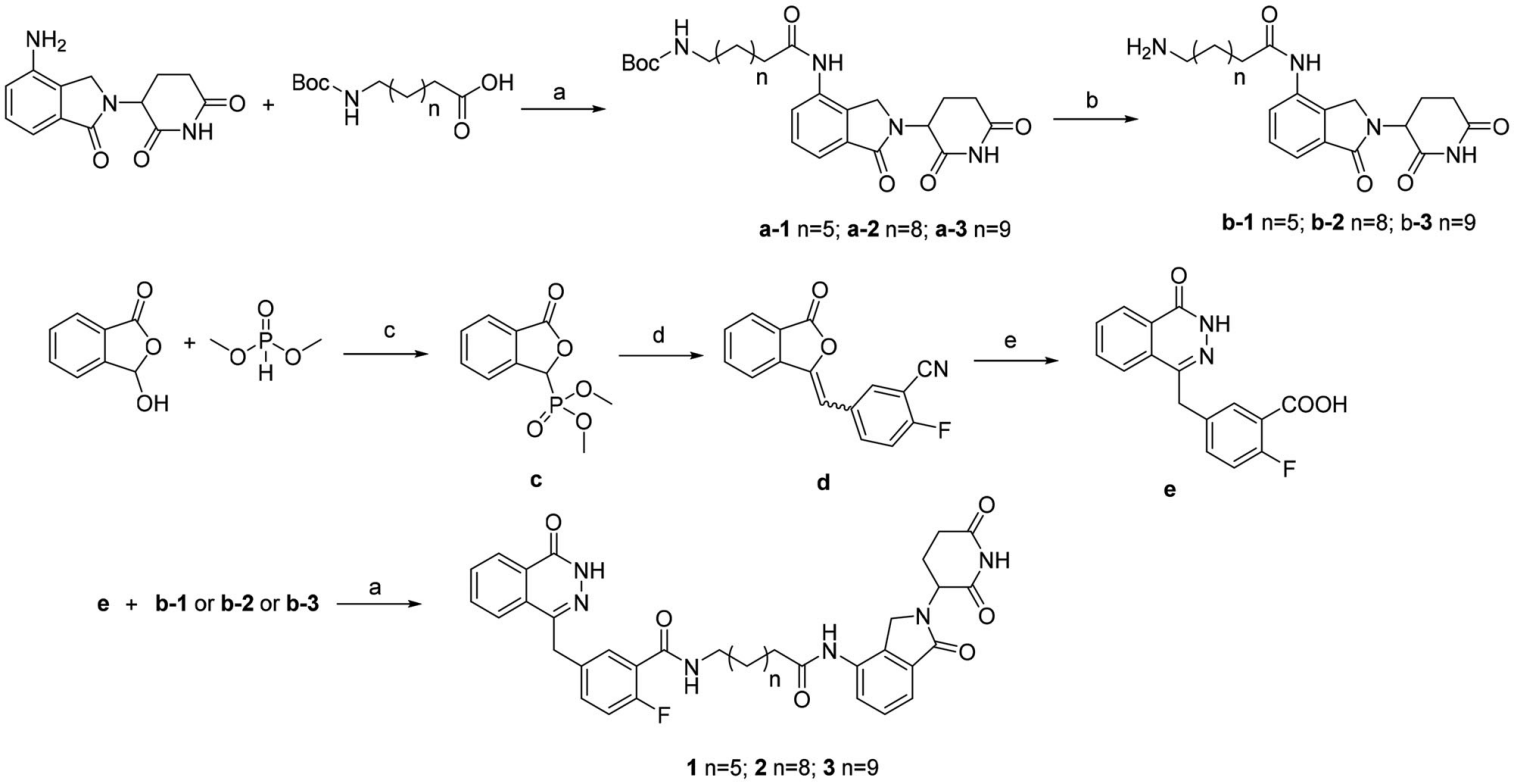
Synthetic route to compounds **1–3**. Reagents and conditions: (a) HATU, Et_3_N, DMF; (b) TFA, CH_2_Cl_2_, 4 h, 77%; (c) MeONa, MeOH, 0–25 °C, 89%; (d) 2-fluoro-5-formylbenzonitrile, TEA, THF, 10–20 °C, 84%; (e) i. NaOH, H_2_O, 90 °C. ii. N_2_H_2_·H_2_O, 70 °C. iii. 2 M HCl, H_2_O, r. t. 73%.

The syntheses of the presented final compounds were outlined in Figure 3. Compound **a-1** was carried out by the reaction of commercially available 8-((tert-butoxycarbonyl)amino)octanoic acid and lenalidomide in the presence of HATU (a polypeptide condensation reagent) and trimethylamine. Boc deprotection of **a-1** led to the key intermediate **b-1**. Compounds **a-2** and **a-3** were synthesised using the procedure described for the synthesis of compound **a-1.** The synthesis process began with the displacement of the commercially available dimethyl phosphite to *o*-phthalaldehydic acid and generated the corresponding phosphonate **c** in 89% yield. Addition of 2-fluoro-5-formylbenzonitrile to **c** led to the formation of benzalphthalide **d** in 84% yield as a mixture of *E/Z* isomers. The mixture of *E* and *Z* isomers were treated with hydrazine hydrate to produce the phthalazinone core. Base hydrolysis of the pendant nitrile provided the second key carboxylic acid intermediate **e**. The final compounds **1–3** were obtained by coupling of **e** and **b-1–b-3** under HATU condition in 45–53% yield.

### Evaluation of the degradation profile on PARPP-1

**Figure 4.**
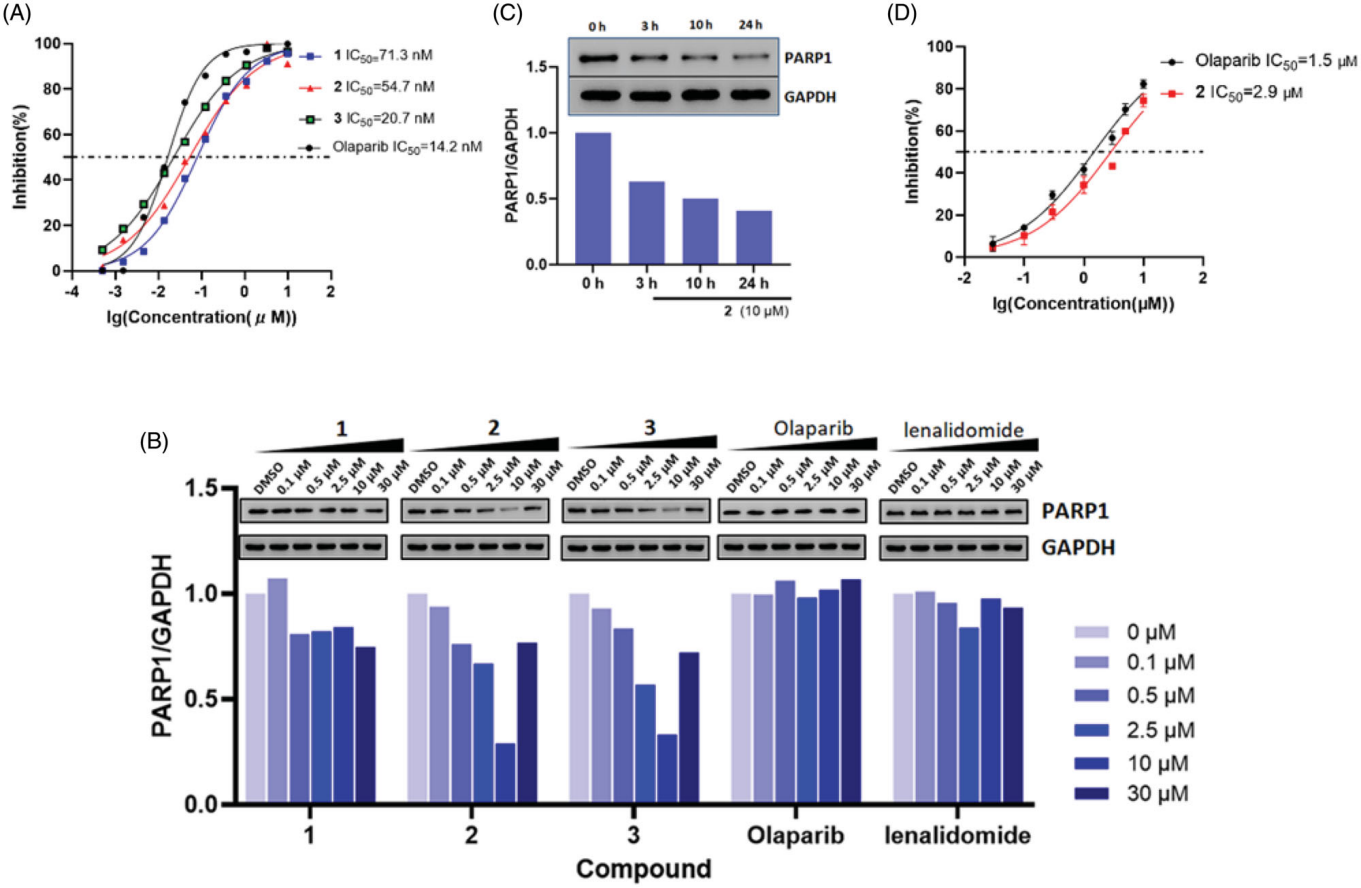
(A) Dose-response curves of compound **1–3** and olaparib for PARP-1 inhibition. (B) Dose-dependent western blot analysis of PARP-1 and GAPDH after treatment of SW620 cells with six different concentrations of compounds **1**, **2**, **3**, olaparib and lenalidomide for 24 h before harvesting. (C) Time-dependent experiment in SW620 cells after treatment with 10 μM compound **2** at the desired time points. (D) Dose-response curves of compound **2** and olaparib in incubation with cancer cell lines (mean ± SD, *n* = 3).

With these PROTAC based compounds in hand, we next investigated their ability to inhibit PARP-1 in a cell-free system *in vitro*. As shown in Figure 4(A), compound **1–3** exhibited slightly weaker cell-free PARP-1 inhibition activities (IC_50_ = 71.3, 54.7, 20.7 nM) compared to that of olaparib (IC_50_ = 14.2 nM) confirming that structural variance of olaparib had a minor influence on PARP-1 inhibition potency. Although these PROTACs showed decreased PARP-1 inhibition, we speculate that their ability of PARP-1 degradation would not be greatly affected. On account of the event-driven modality of PROTACs, the degradability of PROTACs is independent of affinity to target proteins.

While this research was underway, Yu Rao and co-workers successfully disclosed the first PARP-1 degrader based on a niraparib derivative which induces PARP-1 cleavage and cell apoptosis in the MDA-MB-231 cell line^33^. Here, we chose colorectal adenocarcinoma SW620 cell line that highly expressed PARP-1^14^ to prove the suitability of our olaparib-based PROTAC **1–3** for chemically induced PARP-1 degradation. Western blotting data (Figure 4(B)) showed that compound **2** and **3**, with longer linker sizes of eleven and twelve carbon atoms, more potently reduced the PARP-1 protein levels in SW620 cells compared to compound **1** with the short linker of eight carbon atoms. This is in line with the extensive reports that the length of linkers in PROTACs has a major effect on their protein degradation^34^. In SW620 cell lines, compound **2** and **3** induced the PARP-1 degradation with DC_50_ (50% degradation) values of 5.4 μM and 6.2 μM after 24 h incubation. The decrease in PARP-1 protein levels was observed after 3 h and reached its maximum at 24 h (Figure 4(C)). After treating with 10 μM of compound **2**, nearly 80% reduction of the PARP-1 levels was achieved. By contrast, neither olaparib nor lenalidomide were observed protein degradation. Furthermore, the “hook effect” was also observed on compound **2** treatment at 30 μM. As a consequence, higher concentrations of compound were not executed in the blots.

Because **2** was the most potent inducer of PARP-1 degradation, we tested its impact on cell viability with the CCK-8 assay. In the range of 0.03–10 μM, we were able to show a concentration-dependent effect of compound **2** and olaparib in inhibiting SW620 cancer cells proliferation. Treatment with compound **2** at 10 μM, inhibited the cell growth by nearly 75% at 48 h, which is comparable to that of olaparib. The IC_50_ values of **2** and olaparib were 2.9 and 1.5 μM, respectively (Figure 4(D)). The result was not in line with our expectations that PARP-1 degraders were more potent than PARP-1 inhibitors in inhibiting cell growth under same concentrations. The basis for the slight weaker cancer cell inhibition activity of our degrader may be due to the poor cell membranes permeability. An in-depth study of the improvement of inhibition activity should conduct in our future research. We considered to replace the alkyl linker by the PEG linker, because the PEG linkers had better metabolic stability in previous researches^35^.

### Effect of 2 on cell apoptosis

**Figure 5.**
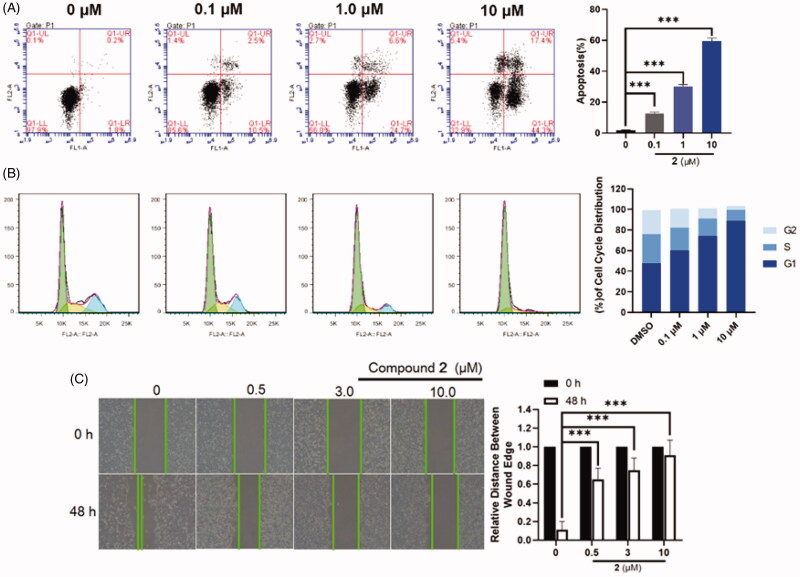
(A) Flow cytometry analysis of apoptosis induction by compound **2** in SW620 cells at the indicated concentrations for 24 h. Data are expressed as mean ± SD of three independent experiments. Significant differences between each of the indicated groups were determined using the Student’s *t* test. *p* < 0.001 (***). (B) Effects of compound **2** on cell cycle arrest at the indicated concentrations for 48 h. Data are obtained through three independent experiments. (C) SW620 cells were treated with compound **2** (0–10.0 μM) for 48 h. Afterwards, photographs were captured, these results are summarised in right. Data are expressed as mean ± SD of three independent experiments. Significant differences between each of the indicated groups were determined using the Student’s *t* test. *p* < 0.001 (***).

As is well-known, the cleavage of PARP-1 is a marker for cell apoptosis^33^^,^^36^, we speculate that PARP-1 degradation induced by compound **2** in SW620 cells may induce cell apoptosis. To further explore its anti-proliferative mechanism, flow cytometry analysis was applied to evaluate the effect of compound **2** on apoptosis. It was found that compound **2** was effectively induced apoptosis in a dose-dependent manner and preliminarily induced apoptosis at a concentration as low as 0.1 μM upon a 24 h treatment (Figure 5(A)). When the dose was increased to 10 μM, the apoptosis ratios of compound **2** were up to 60%. Furthermore, the ability of the compound **2** in inducing cell cycle arrest was explored in SW620 cell lines through flow cytometry analysis. SW620 cells were incubated with 0.1 μM, 1.0 μM, and 10 μM concentrations of compound **2** for 24 h with DMSO as the negative control. The results (Figure 5(B)) suggested that the cell cycle spectrum clearly changed in a dose-dependent manner. However, our result suggested that our degrader arrested the cell-cycle into the G1 phase, while PARP-1 inhibitors generally arrested the cell-cycle into the G2/M phase^37^. The facts that they arrested the cell-cycle into the different phases was attributed to the different mechanisms of inhibitors and degraders. Inhibitors resulted in the discontinuation of DNA replication, due to the inability of PARP-1 to fall off the DNA damage site. The PROTAC directly degraded the PARP-1 protein, which caused that PARP-1 lost the ability to repair DNA damage and thus arrest into the G1 phase. Since compound **2** was very effective at inducing cells apoptosis, we evaluated the effect of **2** on the metastasis of SW620 cancer cells through scratch assays. Compared to the motility of the cells in the control group, the **2**-treated group was markedly lower, suggesting that compound **2** might inhibit the migratory capacity of SW620 cells (Figure 5(C)).

### Evaluation of compound 2 on metabolic-stability

**Figure 6.**
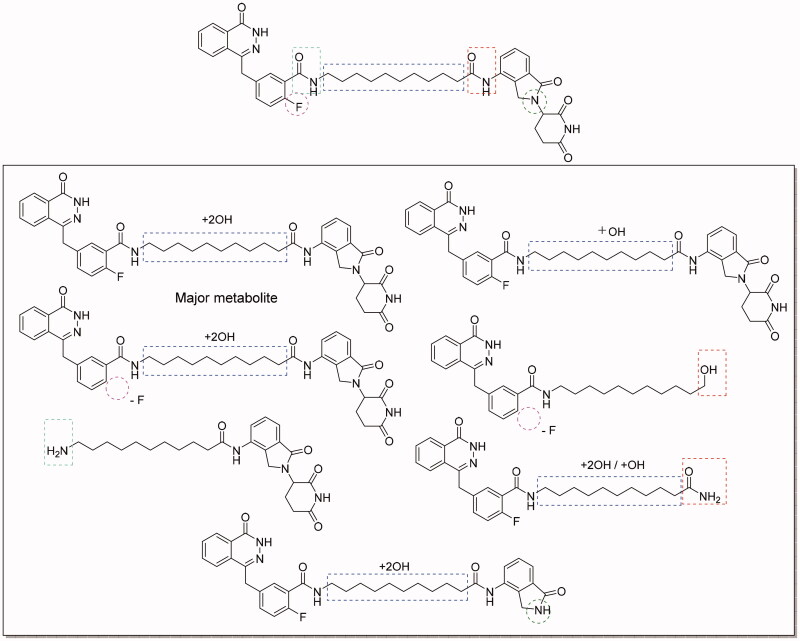
Proposed metabolites of compound **2** in human liver microsomes.

Next, we assessed the preliminary metabolic-stability of compound **2** *in vitro* by using human liver microsomes (HLMs). Compound **2** was incubated with HLMs at 37 °C. Unfortunately, the result indicted that compound **2** was extremely unstable in HLMs. Nearly 90% of compound **2** was metabolised after 30 min incubation and the half-life was only 1.86 min. Then we analysed the proposed metabolites of compound **2** (Figure 6). The major metabolite was proposed to be mono- or di-hydroxylated product occurring in the alkyl chain in the linker. In addition, cleavage of the amide bonds between the linker and cereblon or PARP-1 binding ligand are other alternative metabolic pathways. Metabolic stability is one of the greatest challenges for PROTACs based drug discovery, and our investigation on metabolism locus will provide useful reference for minimising metabolism during the lead compound optimisation. Our next research will focus on improving the metabolic stability by changing the linker instead of the alkyl linkers, such as PEG linkers, alkanoyl linkers, and azide linkers.

## Experimental section

### General chemistry

Commercially available reagents and anhydrous solvents were used without further purification. The crude reaction product was purified by Flash chromatography using silica gel (300–400 mesh). All reactions were monitored by TLC, using silica gel plates with fluorescence F254 and UV light visualisation. If necessary, further purification was performed on a preparative HPLC (Waters 2545) with a C_18_ reverse phase column. Proton nuclear magnetic resonance (^1^H-NMR) and carbon nuclear magnetic resonance (^13^C NMR) spectra were recorded on a Bruker AV-400 spectrometer at 400 MHz. Coupling constants (*J*) are expressed in hertz (Hz). Each signal is identified by its chemical shift δ expressed in parts per million (ppm) HRMS analyzes were performed under ESI (electrospray ionization) using a TOF analyser in V mode with a mass resolution of 9000. The spectrograms of NMR and MS could be found in the Supplementary material.

#### General procedure for the preparation of a-1–a-3

Lenalidomide (2.6 g, 10 mmol) was dissolved in dry DMF (10 mL), a solution of Boc protected amino acid (10 mmol) in dry dimethylformamide (DMF) (2 mL) was then added, followed by the addition of HATU (4.0 g, 10.5 mmol) and TEA (10.5 mmol). The solution was the stirred overnight under argon atmosphere. After the reaction, the solvent was removed under reduced vacuum and without further purified to give crud compound **a-1–a-3** as white solid.

#### General procedure for the preparation of b-1–b-3

Product **a-1–a-3** (1.0 mmol) was stirred with a solution (3 mL) of trifluoroacetic acid TFA/DCM (1:5, v/v) for 2 h. After the reaction, the solution was co-evaporated with toluene (10 mL × 3) under reduced vacuum and further recrystallized from MeOH to get **b-1–b-3** as white solid.

##### 8-amino-*N*-(2–(2,6-dioxopiperidin-3-yl)-1-oxoisoindolin-4-yl)octanamide (b-1)

yield 77%; m.p. 125–127 *°C*. ^1^H NMR (400 MHz, DMSO) δ 11.02 (s, 1H), 9.83 (s, 1H), 7.83 (d, *J* = 6.9 Hz, 2H), 7.78 (s, 1H), 7.49 (q, *J* = 7.3 Hz, 2H), 5.15 (dd, *J* = 13.3, 5.1 Hz, 1H), 4.37 (q, *J* = 17.5 Hz, 2H), 2.98 − 2.87 (m, 1H), 2.78 (dd, *J* = 13.4, 6.7 Hz, 2H), 2.62 (d, *J* = 16.7 Hz, 1H), 2.37 (t, *J* = 7.4 Hz, 3H), 2.09 − 2.00 (m, 1H), 1.57 (d, *J* = 31.9 Hz, 4H), 1.31 (s, 6H). ^13^C NMR (101 MHz, DMSO) δ 173.34, 171.86, 171.55, 168.31, 134.32, 134.09, 133.13, 129.08, 125.64, 119.41, 60.22, 52.00, 46.94, 36.20, 31.67, 28.92, 28.77, 27.44, 26.16, 25.43, 23.11. HRMS m/z: calcd. for C_21_H_29_N_4_O_4_ [M + H]^+^ 401.2229, found 401.2198.

##### 11-amino-*N*-(2–(2,6-dioxopiperidin-3-yl)-1-oxoisoindolin-4-yl)undecanamide(b-2)

yield 84.9%; m.p. 131–133 *°C*. ^1^H NMR (400 MHz, DMSO) δ 11.02 (s, 1H), 9.82 (s, 1H), 7.83 (d, *J* = 7.0 Hz, 2H), 7.79 − 7.72 (m, 1H), 7.53 − 7.44 (m, 2H), 5.15 (dd, *J* = 13.3, 5.1 Hz, 1H), 4.37 (q, *J* = 17.5 Hz, 2H), 3.00 − 2.87 (m, 1H), 2.77 (dd, *J* = 13.9, 6.5 Hz, 2H), 2.62 (d, *J* = 16.8 Hz, 1H), 2.36 (t, *J* = 7.4 Hz, 3H), 2.10 − 1.97 (m, 1H), 1.66 − 1.47 (m, 4H), 1.28 (d, *J* = 11.2 Hz, 12H). ^13^C NMR (101 MHz, DMSO) δ 173.33, 171.90, 171.53, 168.32, 134.33, 134.12, 133.13, 129.07, 125.66, 119.41, 51.99, 46.95, 39.28, 36.25, 31.68, 29.29, 29.27, 29.25, 29.14, 28.97, 27.45, 26.24, 25.56, 23.12. HRMS m/z: calcd. for C_24_H_35_N_4_O_4_ [M + H]^+^ 443.2658, found 443.2659.

##### 12-amino-*N*-(2–(2,6-dioxopiperidin-3-yl)-1-oxoisoindolin-4-yl)dodecanamide (b-3)

yield 84.9%; m.p. 137–139 *°C*. ^1^H NMR (400 MHz, DMSO) δ 11.02 (s, 1H), 9.82 (s, 1H), 7.83 (d, *J* = 7.1 Hz, 2H), 7.79 (s, 2H), 7.55 − 7.44 (m, 2H), 5.15 (dd, *J* = 13.3, 5.1 Hz, 1H), 4.37 (q, *J* = 17.5 Hz, 2H), 2.98 − 2.87 (m, 1H), 2.77 (dd, *J* = 13.7, 6.7 Hz, 2H), 2.62 (d, *J* = 16.9 Hz, 1H), 2.35 (h, *J* = 9.2 Hz, 3H), 2.10 − 1.99 (m, 1H), 1.65 − 1.46 (m, 4H), 1.28 (d, *J* = 13.5 Hz, 14H). ^13^C NMR (101 MHz, DMSO) δ 173.33, 171.91, 171.53, 168.32, 134.32, 134.12, 133.13, 129.07, 125.67, 119.41, 52.00, 46.95, 39.28, 36.25, 31.68, 29.39, 29.35, 29.28, 29.26, 29.13, 28.98, 27.44, 26.24, 25.56, 23.12. HRMS m/z: calcd. for C_25_H_37_N_4_O_4_ [M + H]^+^ 457.2855, found 457.2815.

##### dimethyl (3-oxo-1,3-dihydroisobenzofuran-1-yl)phosphonate (c)

To a 0.5 M solution of sodium methoxide in methanol (19.8 mL, 9.9 mmol) was added dimethyl phosphite (1.3 mL, 14.3 mmol) at 0 °C, and the solution was stirred at 0 °C for 10 min. A suspension of the 3-hydroxyisobenzofuran-1(3*H*)-one (1.62 g, 7.07 mmol) in anhydrous methanol (10 mL) was slowly added and the reaction mixture allowed to warm to room temperature over a period of 1 h. The solution was cooled in an ice-bath, and methanesulfonic acid (1.0 mL, 15.5 mmol) was added dropwise. After the addition, most of the solvent is evaporated under reduced pressure with gentle heating and the residue is partitioned between dichloromethane (100 mL) and cold water (5.0 mL). The organics was washed with brine (50 mL), and concentrated. The residue was dried under vacuum and recrystallized from dichloromethane/ether to yield the title compound. Light yellow solid, yield 89.3%; m.p. 87–88 *°C*. ^1^H NMR (500 MHz, DMSO) δ 7.96 (d, *J* = 7.7 Hz, 1H), 7.88 (t, *J* = 7.5 Hz, 1H), 7.76 − 7.71 (m, 1H), 7.69 (d, *J* = 7.4 Hz, 1H), 6.38 (d, *J* = 11.1 Hz, 1H), 3.84 (d, *J* = 10.9 Hz, 3H), 3.64 (d, *J* = 10.7 Hz, 3H). ^13^C NMR (126 MHz, DMSO) δ 169.88, 144.52, 135.27, 130.39, 125.97, 124.83, 123.95, 75.84, 54.34, 40.01. HRMS m/z: calcd. for C_10_H_12_O_5_P [M + H]^+^ 243.0422, found 243.0420.

##### 2-fluoro-5-((3-oxoisobenzofuran-1(3*H*)-ylidene)methyl)benzonitrile (d)

To a mixture of **c** (3.5 g, 14 mmol) and 2-fluoro-5-formylbenzonitrile (2.1 g, 14 mmol) in THF (50 mL) was added triethylamine (1.4 mL, 14 mmol) dropwise over 25 min, and the temperature was maintained below 20 °C. The reaction mixture was slowly warmed to 20 °C over 1 h and stirred for overnight. The reaction mixture was concentrated and the residue was added to water and stirred for 30 min. The precipitated solid was collected by filtration, washed with water, hexanes, and ether, and dried *in vacuo* to afford the target compound (a mixture of *E* and *Z* isomers), further purified by silica gel column chromatography. White solid, yield 83.6%; m.p. 186–187 °C. ^1^H NMR (500 MHz, DMSO) δ 8.24 − 8.13 (m, 2H), 8.08 (d, *J* = 7.8 Hz, 1H), 8.00 (d, *J* = 7.7 Hz, 1H), 7.93 (t, *J* = 7.6 Hz, 1H), 7.72 (t, *J* = 7.5 Hz, 1H), 7.66 (t, *J* = 9.1 Hz, 1H), 7.00 (d, *J* = 6.3 Hz, 1H). ^13^C NMR (126 MHz, DMSO) δ 166.37, 162.95, 145.89, 140.03, 137.19, 135.84, 134.65, 131.40, 125.82, 123.16, 121.38, 117.87, 114.21, 110.08, 103.88, 101.39. HRMS m/z: calcd. for C_16_H_9_FNO_2_ [M + H]^+^ 266.0617, found 266.0623.

##### 2-fluoro-5-((4-oxo-3,4-dihydrophthalazin-1-yl)methyl)benzoic acid (e)

To a stirred suspension of **d** (3.7 g, 14 mmol) in water (20 mL) was added aqueous sodium hydroxide (2.6 g in 5 mL water) solution and the reaction mixture was heated under nitrogen to 90 °C for 1 h. The reaction mixture was partially cooled to 70 °C and hydrazine hydrate (10 mL, 2.0 mol) added, and the mixture was stirred for 18 h at 70 °C. The reaction was cooled to ambient temperature and was acidified with 2 M HCl to pH 4. The mixture was stirred for 10 min and filtered. The resulting solid was washed with water, followed by diethyl ether and was dried to produce the title compound. White powder. yield 73.1%; m.p. 107–108 *°C*. ^1^H NMR (500 MHz, DMSO) δ 12.65 (s, 1H), 8.30 (d, *J* = 7.9 Hz, 1H), 8.01 (d, *J* = 8.0 Hz, 1H), 7.92 (t, *J* = 7.6 Hz, 1H), 7.86 (t, *J* = 7.0 Hz, 2H), 7.65 − 7.22 (m, 2H), 4.39 (s, 2H). ^13^C NMR (126 MHz, DMSO) δ 165.45, 161.38, 159.83, 145.34, 135.36, 134.76, 133.98, 132.31, 132.01, 129.54, 128.35, 126.54, 125.87, 119.68, 117.53, 36.77. HRMS m/z: calcd. For C_16_H_12_FN2O_3_ [M + H]^+^ 299.0832, found 299.0833.

The preparation of compounds **1–3** followed the procedure of **a-1–a-3**.

##### *N*-(8-((2–(2,6-dioxopiperidin-3-yl)-1-oxoisoindolin-4-yl)amino)-8-oxooctyl)-2-fluoro-5-((4-oxo-3,4-dihydrophthalazin-1-yl)methyl)benzamide (1)

White powder. yield 52.7%; m.p. 141–143 *°C*. ^1^H NMR (500 MHz, DMSO-d6) δ 12.65 (s, 1H), 11.06 (s, 1H), 9.96 (s, 1H), 8.29 (d, *J* = 7.9 Hz, 2H), 7.98 (d, *J* = 4.3 Hz, 1H), 7.90 (d, *J* = 7.1 Hz, 1H), 7.83 (dd, *J* = 13.5, 6.4 Hz, 2H), 7.57 (d, *J* = 6.7 Hz, 1H), 7.52 (dd, *J* = 13.0, 7.5 Hz, 2H), 7.23 − 7.18 (m, 1H), 5.18 (dd, *J* = 13.3, 5.1 Hz, 1H), 4.34 (s, 2H), 3.24 (dd, *J* = 12.9, 6.6 Hz, 2H), 2.90 (s, 1H), 2.70 (s, 3H), 2.40 (t, *J* = 7.4 Hz, 2H), 1.94 (s, 2H), 1.65 − 1.48 (m, 4H), 1.33 (s, 6H). ^13^C NMR (126 MHz, DMSO-d6) δ 173.35, 172.52, 171.97, 171.54, 168.36, 163.94, 159.89, 157.28, 145.44, 134.74, 134.34, 133.97, 133.11, 132.71, 132.00, 130.40, 129.51, 129.03, 128.33, 126.52, 125.97, 125.72, 124.81, 119.38, 116.66, 52.04, 38.68, 36.93, 36.25, 31.67, 30.87, 29.34, 29.10, 26.73, 25.54, 23.12, 21.56. HRMS m/z: calcd. for C_37_H_38_FN_6_O_6_ [M + H]^+^ 681.2837, found 681.2838.

##### *N*-(11-((2–(2,6-dioxopiperidin-3-yl)-1-oxoisoindolin-4-yl)amino)-11-oxoundecyl)-2-fluoro-5-((4-oxo-3,4-dihydrophthalazin-1-yl)methyl)benzamide (2)

White powder. yield 45.4%; m.p. 130–131 °C. ^1^H NMR (500 MHz, DMSO-d6) δ 12.62 (s, 1H), 11.05 (s, 1H), 9.78 (s, 1H), 8.28 (d, *J* = 7.8 Hz, 1H), 8.25 (s, 1H), 7.97 (d, *J* = 7.9 Hz, 1H), 7.89 (t, *J* = 8.3 Hz, 1H), 7.83 (t, *J* = 8.2 Hz, 2H), 7.55 (d, *J* = 6.8 Hz, 1H), 7.51 (d, *J* = 5.3 Hz, 1H), 7.47 − 7.42 (m, 1H), 7.23 − 7.17 (m, 1H), 5.18 (dd, *J* = 13.3, 5.1 Hz, 1H), 4.33 (s, 2H), 3.40 (s, 3H), 3.22 (dd, *J* = 13.0, 6.7 Hz, 2H), 2.99 − 2.90 (m, 1H), 2.63 (d, *J* = 17.0 Hz, 1H), 2.36 (d, *J* = 7.6 Hz, 2H), 2.00 (s, 1H), 1.63 − 1.46 (m, 4H), 1.28 (d, *J* = 15.4 Hz, 12H). ^13^C NMR (126 MHz, DMSO-d6) δ 173.33, 171.88, 171.55, 168.33, 163.89, 159.87, 157.29, 145.40, 134.77, 134.29, 134.12, 133.95, 133.13, 132.72, 131.99, 130.43, 129.53, 129.07, 128.36, 126.53, 125.96, 125.68, 124.81, 119.43, 116.66, 60.22, 55.36, 52.01, 46.95, 36.94, 36.30, 31.69, 29.44, 29.37, 29.16, 26.83, 25.57, 23.13, 21.21, 14.54. HRMS m/z: calcd. for C_40_H_44_FN_6_O_6_ [M + H]^+^ 723.3306, found 723.3298.

##### *N*-(12-((2–(2,6-dioxopiperidin-3-yl)-1-oxoisoindolin-4-yl)amino)-12-oxododecyl)-2-fluoro-5-((4-oxo-3,4-dihydrophthalazin-1-yl)methyl)benzamide (3)

White powder. yield 50.6%; m.p. 132–133 *°C*. ^1^H NMR (500 MHz, DMSO-d6) δ 12.61 (s, 1H), 11.04 (s, 1H), 9.77 (s, 1H), 8.27 (d, *J* = 7.8 Hz, 1H), 8.24 (t, *J* = 4.7 Hz, 1H), 7.97 (d, *J* = 7.9 Hz, 1H), 7.88 (t, *J* = 7.6 Hz, 1H), 7.86 − 7.78 (m, 2H), 7.54 (dd, *J* = 6.8, 2.3 Hz, 1H), 7.51 − 7.50 (m, 1H), 7.47 − 7.41 (m, 1H), 7.25 − 7.14 (m, 1H), 5.16 (dd, *J* = 13.3, 5.1 Hz, 1H), 4.33 (s, 2H), 3.37 (s, 4H), 3.20 (dd, *J* = 13.0, 6.7 Hz, 2H), 2.98 − 2.88 (m, 1H), 2.62 (d, *J* = 16.9 Hz, 1H), 2.37 − 2.34 (m, 2H), 1.63 − 1.45 (m, 4H), 1.31 − 1.23 (m, 14H). ^13^C NMR (126 MHz, DMSO-d6) δ 173.33, 171.87, 171.55, 168.32, 163.88, 159.86, 157.27, 145.41, 134.77, 134.29, 134.13, 133.97, 133.13, 132.72, 132.01, 130.42, 129.53, 129.08, 128.36, 126.53, 125.98, 125.69, 124.82, 119.43, 116.66, 60.23, 55.38, 54.07, 52.00, 46.94, 36.93, 36.29, 31.68, 29.45, 29.42, 29.37, 29.26, 29.18, 26.82, 25.56, 23.13. HRMS m/z: calcd. for C_41_H_46_FN_6_O_6_ [M + H]^+^ 737.3463, found 737.3452.

### Cell culture

Colorectal cancer SW620 cells were purchased from the Cell Centre of the Chinese Academy of Medical Sciences (Beijing, China). First passages were carried out in Leibovitz medium; the Leibovitz medium was then gradually substituted with Dulbeccos’s modified Eagle medium (DMEM). This contained 25 mmol/L glucose and 2 mmol/L l-glutamine, 10% horse serum, 100 U/mL penicillin, and 100 mg/mL streptomycin. Incubations were carried out at 37 °C in a humified atmosphere of 5% CO_2_. The culture medium was changed every 48 h.

### Western blot analysis

To determine levels of PARP-1, cells were seeded in a 6-well cell culture plate at a density of 400 000 cells per well for SW620 in a total volume of 1800 μL and incubated overnight in L15 medium containing 10% foetal bovine serum (Life Technologies, Rockville, MD). Then 200 μL of serially diluted compounds were added to each well the next day. Cell lysates were harvested after 48 h and PARP-1 were quantified using assay kits following the manufacturer’s protocols. Rabbit monoclonal antibody for PARP-1 was from Cell Signalling Technology (Danvers, MA, USA).

### Anti-proliferative assay

Colorectal cancer SW620 were seeded in 96-well plates at a density of 4000–5000 cells per well. Cells were allowed to adhere for 12 h and starved with serum-free medium for additional 12 h. Medium containing a certain concentration of compound was added into each well in a volume of 100 μL for 24 h respectively. CCK8 (Zomanbio, China) was added to each well and incubated for 1 h at 37 °C. The optical density (OD) was measured at a wavelength of 450 nm using a microplate reader.

### Migration assay

SW620 cells were cultured in 6-well dish plates at a number of 3 × 10^5^ cells/well and grown overnight to confluence. A wound was created by scratching a straight line in the monolayer with a 200 μL pipet tip. The cells were then incubated with compound **2** in serum-free medium for 4836 h and the wound area was then photographed. The rate of wound closure was assessed by measuring distances from six randomly selected fields.

### Flow cytometry

SW620 cells were seeded in 6-well dish plates at 1 × 10^5^ cells/well and exposed to different concentrations of compound **2**. After 24 h, the cells were harvested and stained with Annexin-V/PI Solution using the FITC Annexin V Apoptosis Detection Kit I (BD Bioscience) for 10–15 min and resuspended in binding buffer. Then the fluorescence emission at 530 nm and 585 nm using 488 nm excitation was measured by flow cytometry (BD FACSCalibur).

### Liver microsome stability assay

The metabolic stability was assessed using human liver microsomes (purchased from Ltd Co (RILD), M008084). Briefly, 1 μM of compound **2** was incubated with 1.7 mM cofactor β-NADPH and 0.5 mg/mL microsomes in 0.1 M phosphate buffer (pH = 7.4) containing 3.3 mM MgCl_2_ at 37 °C. The DMSO concentration was less than 0.1% in the final incubation system. At 0.025, 0.083, 0.25, 0.5, 1.0, 1.5 and 2 h of incubation, an amount of 60 μL of reaction mixture was taken out, and the reaction is stopped immediately by adding 3-fold excess of cold acetonitrile containing 100 ng/mL of internal standard for quantification. The collected fractions were centrifuged at 30 000 rpm for 5 min to collect the supernatant for LC − MS/MS analysis, from which the amount of compound remaining was determined.

## Conclusion

In this study, we report our design, synthesis, and evaluation of the PROTAC small-molecule PARP-1 degrader probes. We thoroughly characterise compound **2** as a novel and effective PARP-1 degrader although its potency and metabolic stability are still remained to further improve. These are also the mainly problems that our next research eager to solve. Biological and mechanistic studies suggest that compound **2** starts to induce PARP-1 degradation at the concentration lower than 100 nM in SW620 cell lines with 24 h treatment and is capable of achieving nearly 80% PARP-1 degradation in this cell line. Compound **2** potently inhibits SW620 cell growth which is comparable to olaparib. Moreover, compound **2** also significantly arrested the cell cycle distribution and induced cell apoptosis. Our study thus qualify compound **2** as a novel chemical probe that will be valuable to explore the biology and therapeutic potential of PARP-1 degradation. The structure optimisation study of compound **2** is underway in our laboratory.

## Supplementary Material

Supplemental Material

## References

[1] Ding J, Miao ZH, Meng LH, Geng MY. Emerging cancer therapeutic opportunities target DNA-repair systems. Trends Pharmacol Sci 2006;27:338–44. 16697054 10.1016/j.tips.2006.04.007

[2] Gibson BA, Kraus WL. New insights into the molecular and cellular functions of poly(ADP-ribose) and PARPs. Nat Rev Mol Cell Biol 2012;13:411–24. 22713970 10.1038/nrm3376

[3] D’amours D, Desnoyers S, D’silva I, Poirier GG. Poly(ADP-ribosyl)ation reactions in there gulation of nuclear functions. Biochem J 1999;342: 249–68. 10455009 PMC1220459

[4] Morales JC, Li LS, Fattah FJ, et al. Review of poly (ADP-ribose) polymerase (PARP) mechanisms of action and rationale for targeting in cancer and other diseases. Crit Rev Eukaryot Gene Expr 2014;24:15–28. 24579667 10.1615/critreveukaryotgeneexpr.2013006875 PMC4806654

[5] Huber A, Bai P, de Murcia JM, de Murcia G. PARP-1, PARP-2 and ATM in the DNA damage response: functional synergy in mouse development. DNA Repair 2004;3:1103–8. 15279798 10.1016/j.dnarep.2004.06.002

[6] Gao CZ, Dong W, Cui ZW, et al. Synthesis, preliminarily biological evaluation and molecular docking study of new Olaparib analogues as multifunctional PARP-1 and cholinesterase inhibitors. J Enzyme Inhib Med Chem 2019;34:150–62. 30427217 10.1080/14756366.2018.1530224 PMC6237161

[7] Wang ZQ, Auer B, Stingl L, et al. Mice lacking ADPRT and poly(ADP-ribosyl)ation develop normally but are susceptible to skin disease. Genes Dev 1995;9:509–20. 7698643 10.1101/gad.9.5.509

[8] Wang YQ, Wang PY, Wang YT, et al. An update on Poly(ADP-ribose)polymerase-1 (PARP-1) inhibitors: opportunities and challenges in cancer therapy. J Med Chem 2016;59:9575–98. 27416328 10.1021/acs.jmedchem.6b00055

[9] Ferraris DV. Evolution of poly(ADP-ribose) polymerase-1 (PARP-1) inhibitors. From concept to clinic. J Med Chem 2010;53:4561–84. 20364863 10.1021/jm100012m

[10] Vyas S, Chang P. New PARP targets for cancer therapy. Nat Rev Cancer 2014;14:502–9. 24898058 10.1038/nrc3748 PMC4480224

[11] He JX, Yang CH, Miao ZH. Poly(ADP-ribose) polymerase inhibitors as promising cancer therapeutics. Acta Pharmacol Sin 2010;31:1172–80. 20676117 10.1038/aps.2010.103 PMC4002295

[12] O’Shaughnessy J, Osborne C, Pippen JE, et al. Iniparib plus chemotherapy in metastatic triple-negative breast cancer. N Engl J Med 2011;364:205–14. 21208101 10.1056/NEJMoa1011418

[13] O’Shaughnessy J, Schwartzberg L, Danso MA, et al. Phase III study of iniparib plus gemcitabine and carboplatin versus gemcitabine and carboplatin in patients with metastatic triple-negative breast cancer. J Clin Oncol 2014;32:3840–7. 25349301 10.1200/JCO.2014.55.2984

[14] Menear KA, Adcock C, Boulter R, et al. 4-[3-(4-cyclopropanecarbonylpiperazine-1-carbonyl)-4-fluorobenzyl]-2H-phthalazin-1-one: a novel bioavailable inhibitor of poly(ADP-ribose) polymerase-1. J Med Chem 2008;51:6581–91. 18800822 10.1021/jm8001263

[15] Thomas HD, Calabrese CR, Batey MA, et al. Preclinical selection of a novel poly(ADP-ribose) polymerase inhibitor for clinical trial. Mol Cancer Ther 2007;6:945–56. 17363489 10.1158/1535-7163.MCT-06-0552

[16] Jones P, Wilcoxen K, Rowley M, Toniatti C. Niraparib: a Poly(ADP-ribose) Polymerase (PARP) inhibitor for the treatment of tumors with defective homologous recombination. J Med Chem 2015;58:3302–14. 25761096 10.1021/jm5018237

[17] Zhou JX, Feng LJ, Zhang X. Risk of severe hematologic toxicities in cancer patients treated with PARP inhibitors: a meta-analysis of randomized controlled trials. Drug Des Devel Ther 2017;11:3009–17. 10.2147/DDDT.S147726 PMC5648323 29075104

[18] Liu Y, Meng J, Wang G. Risk of selected gastrointestinal toxicities associated with poly (ADP-ribose) polymerase (PARP) inhibitors in the treatment of ovarian cancer: a meta-analysis of published trials. Drug Des Devel Ther 2018;Volume 12:3013–9. 10.2147/DDDT.S164553 PMC6147204 30271116

[19] Toure M, Crews CM. Small-molecule PROTACS: new approaches to protein degradation. Angew Chem Int Ed Engl 2016;55:1966–73. 26756721 10.1002/anie.201507978

[20] Ottis P, Crews CM. Proteolysis-targeting chimeras: induced protein degradation as a therapeutic strategy. ACS Chem Biol 2017;12:892–8. 28263557 10.1021/acschembio.6b01068

[21] Churcher I. Protac-induced protein degradation in drug discovery: breaking the rules or just making new ones? J Med Chem 2018;61:444–52. 29144739 10.1021/acs.jmedchem.7b01272

[22] Lai AC, Crews CM. Induced protein degradation: an emerging drug discovery paradigm. Nat Rev Drug Discov 2017;16:101–14. 27885283 10.1038/nrd.2016.211 PMC5684876

[23] Xi M, Chen Y, Yang H, et al. Small molecule PROTACs in targeted therapy: an emerging strategy to induce protein degradation. Eur J Med Chem 2019;174:159–80. 31035238 10.1016/j.ejmech.2019.04.036

[24] Zhang C, Han XR, Yang X, et al. Proteolysis targeting chimeras (PROTACs) of Anaplastic Lymphoma Kinase (ALK). Eur J Med Chem 2018;151:304–14. 29627725 10.1016/j.ejmech.2018.03.071 PMC5924614

[25] Lu M, Liu T, Jiao Q, et al. Discovery of a Keap1-dependent peptide PROTAC to knockdown Tau by ubiquitination-proteasome degradation pathway. Eur J Med Chem 2018;146:251–9. 29407955 10.1016/j.ejmech.2018.01.063

[26] Wang B, Wu S, Liu J, et al. Development of selective small molecule MDM2 degraders based on nutlin. Eur J Med Chem 2019;176:476–91. 31128449 10.1016/j.ejmech.2019.05.046

[27] Li W, Gao C, Zhao L, et al. Phthalimide conjugations for the degradation of oncogenic PI3K. Eur J Med Chem 2018;151:237–47. 29625382 10.1016/j.ejmech.2018.03.066

[28] Han X, Wang C, Qin C, et al. Discovery of ARD-69 as a Highly Potent Proteolysis Targeting Chimera (PROTAC) Degrader of Androgen Receptor (AR) for the Treatment of Prostate Cancer. J Med Chem 2019;62:941–64. 30629437 10.1021/acs.jmedchem.8b01631

[29] Hu J, Hu B, Wang M, et al. Discovery of ERD-308 as a Highly Potent Proteolysis Targeting Chimera (PROTAC) Degrader of Estrogen Receptor (ER). J Med Chem 2019;62:1420–42. 30990042 10.1021/acs.jmedchem.8b01572

[30] Qin C, Hu Y, Zhou B, et al. Discovery of QCA570 as an Exceptionally Potent and Efficacious Proteolysis Targeting Chimera (PROTAC) Degrader of the Bromodomain and Extra-Terminal (BET) proteins capable of inducing complete and durable tumor regression. J Med Chem 2018;61:6685–704. 30019901 10.1021/acs.jmedchem.8b00506 PMC6545111

[31] Schiedel M, Herp D, Hammelmann S, et al. Chemically Induced Degradation of Sirtuin 2 (Sirt2) by a Proteolysis Targeting Chimera (PROTAC) Based on Sirtuin Rearranging Ligands (SirReals). J Med Chem 2018;61:482–91. 28379698 10.1021/acs.jmedchem.6b01872

[32] Edmondson SD, Yang B, Fallan C. Proteolysis targeting chimeras (PROTACs) in ‘beyond rule-of-five’ chemical space: recent progress and future challenges. Bioorg Med Chem Lett 2019;29:1555–64. 31047748 10.1016/j.bmcl.2019.04.030

[33] Zhao Q, Lan T, Su S, Rao Y. Induction of apoptosis in MDA-MB-231 breast cancer cells by a PARP1-targeting PROTAC small molecule. Chem Commun 2019;55:369–72. 10.1039/c8cc07813k 30540295

[34] Gadd MS, Testa A, Lucas X, et al. Structural basis of PROTAC cooperative recognition for selective protein degradation. Nat Chem Biol 2017;13:514–21. 28288108 10.1038/nchembio.2329 PMC5392356

[35] Webster R, Didier E, Harris P, et al. PEGylated proteins: evaluation of their safety in the absence of definitive metabolism studies. Drug Metab Dispos 2007;35:9–16. 17020954 10.1124/dmd.106.012419

[36] Germain M, Affar EB, D’Amours D, et al. Cleavage of automodified poly(ADP-ribose) polymerase during apoptosis. evidence for involvement of caspase-7. J Biol Chem 1999;274:28379–84. 10497198 10.1074/jbc.274.40.28379

[37] Guo C, Wang L, Li X, et al. Discovery of novel bromophenol-thiosemicarbazone hybrids as potent selective inhibitors of Poly(ADP-ribose) polymerase-1 (PARP-1) for use in cancer. J Med Chem 2019;62:3051–67. 30844273 10.1021/acs.jmedchem.8b01946

