# Supplementary material for: Identification of probe-quality degraders for Poly(ADP-ribose) polymerase-1 (PARP-1)
Source: J Enzyme Inhib Med Chem. 2020 Aug 11;35(1):1606–15. doi: 10.1080/14756366.2020.1804382 (PMC7470090; doi:10.1080/14756366.2020.1804382)

Supporting Information

Identification of probe-quality degraders for Poly (ADP-ribose) polymerase-1 (PARP-1)

Zhimin Zhang,[a, #] Xinyue Chang,[a, #] Chixiao Zhang,[a] Shenxin Zeng,[a] Meihao Liang,[a] Zhen Ma,[a] Zunyuan Wang[a] and Wenhai Huang,[a,*] Zhengrong Shen[a,*]

[a]*Key Laboratory of Neuropsychiatric Drug Research of Zhejiang Province, Hangzhou Medical College, Hangzhou, 310013, P. R. China*

[#]*These authors contributed equally and should be considered as co-first authors*

Correspondence

Zhengrong Shen, Key Laboratory of Neuropsychiatric Drug Research of Zhejiang Province, Hangzhou Medical College, Hangzhou 310013, P. R. China Tel.: +86 571 88215506; fax: +86 571 88215625.

E-mail address: shenzr@zjams.com.cn

Wenhai Huang, Key Laboratory of Neuropsychiatric Drug Research of Zhejiang Province, Hangzhou Medical College, Hangzhou 310013, P. R. China Tel.: +86 13857191918; E-mail address: cyj@zju.edu.cn

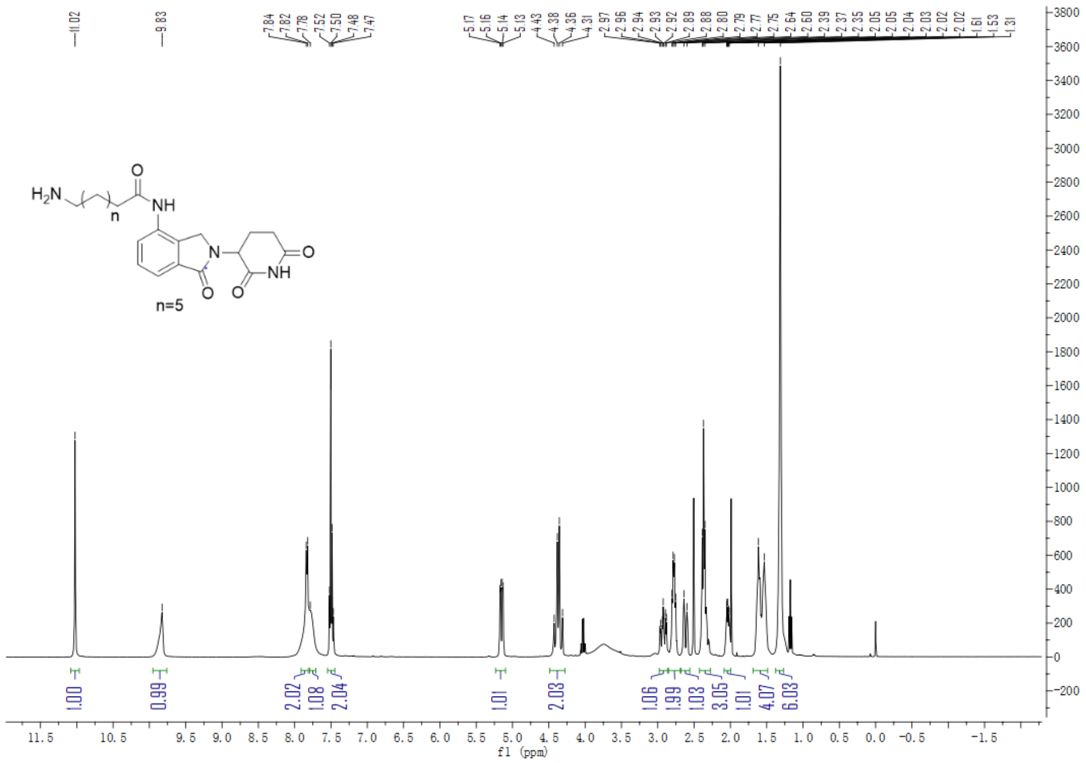

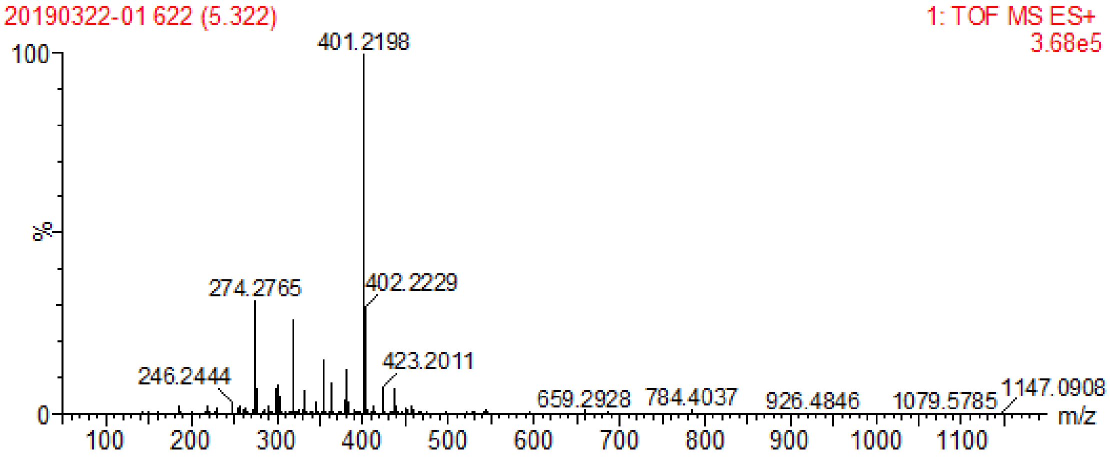
20190322-01 622 (5.322)
1: TOF MS ES+
3.68e5
401.2198
274.2765
402.2229
246.2444
423.2011
659.2928
784.4037
926.4846
1079.5785
1147.0908
m/z

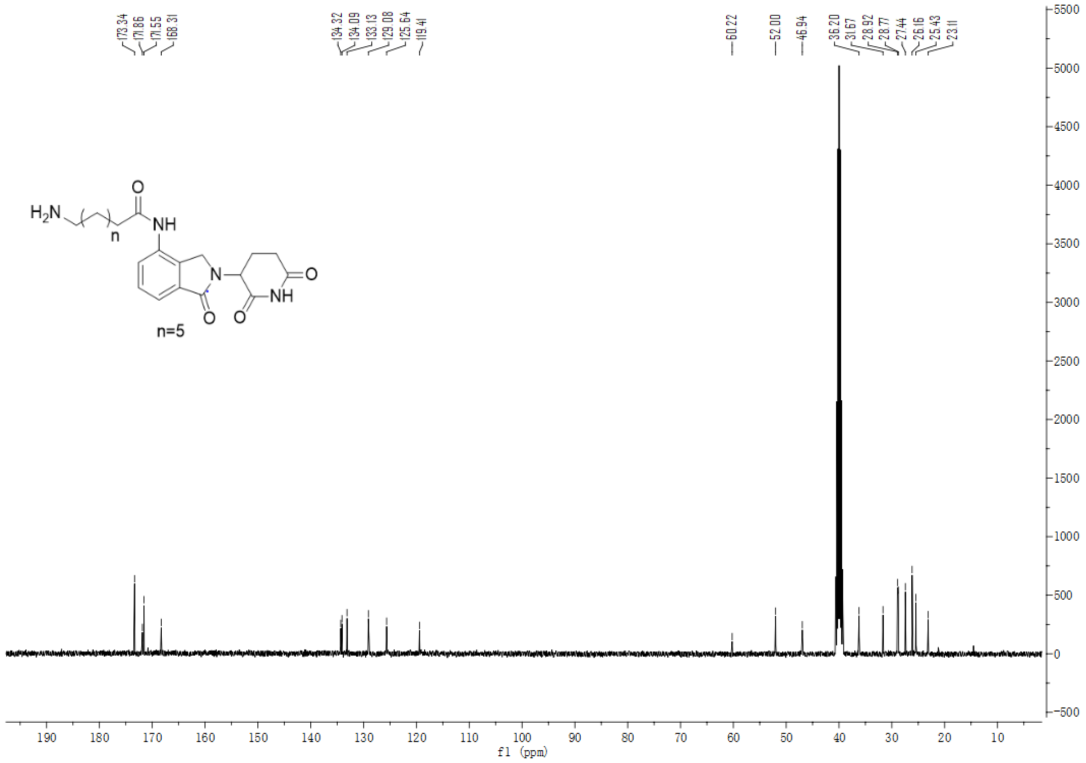
173.34
171.86
171.55
168.31
134.32
134.09
133.13
129.08
125.64
119.41
60.22
52.00
46.94
36.20
31.67
28.92
28.77
27.44
26.16
25.43
23.11
n=5
f1 (ppm)

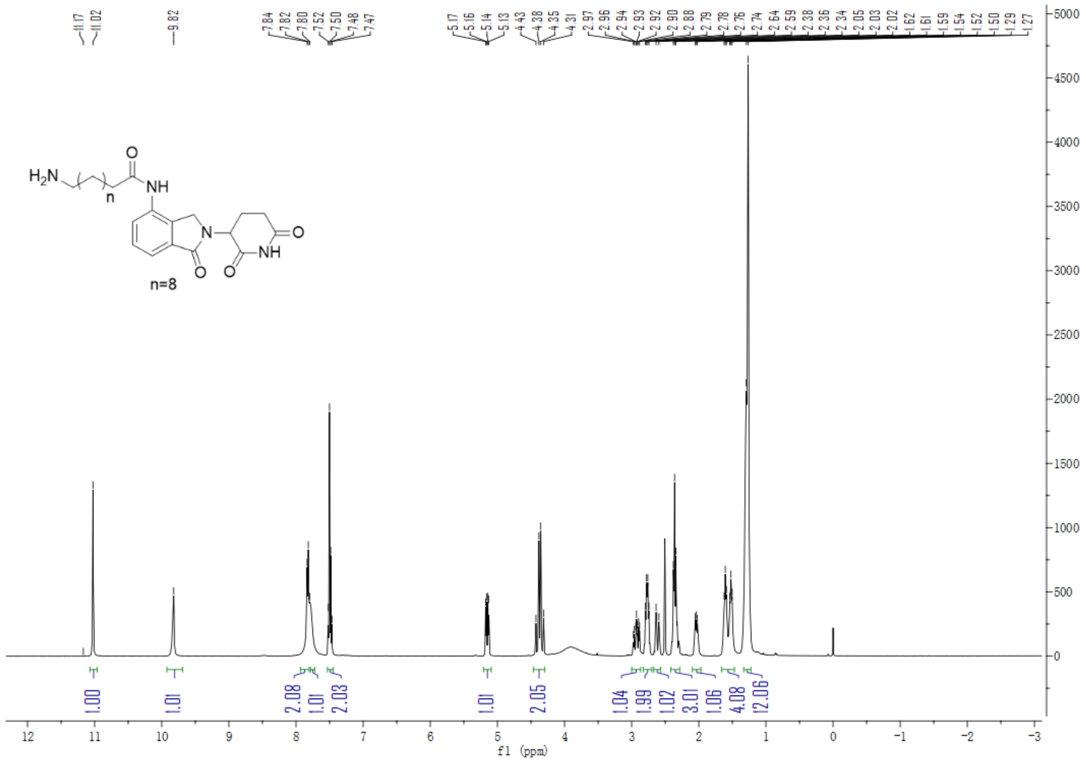

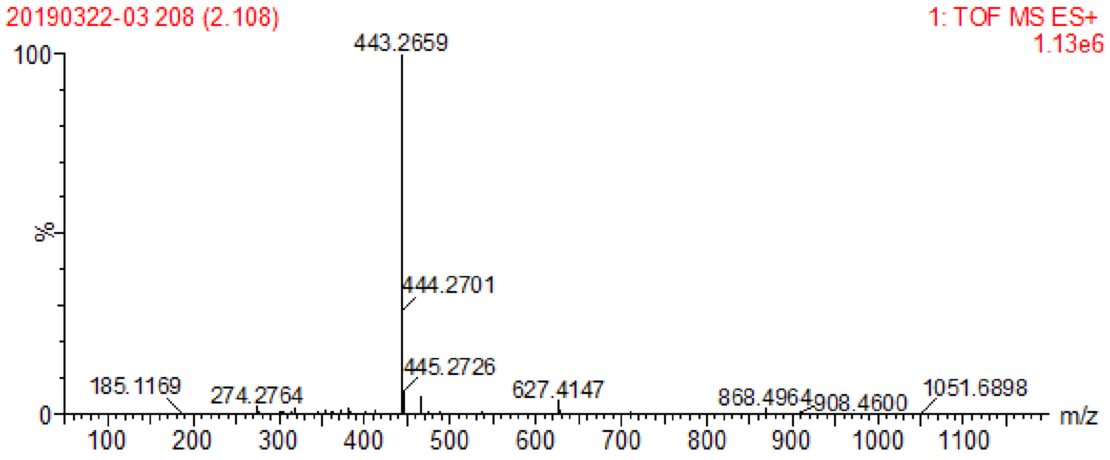

20190322-03 208 (2.108)
1: TOF MS ES+
1.13e6
443.2659
444.2701
445.2726
185.1169
274.2764
627.4147
868.4964
908.4600
1051.6898
m/z
%

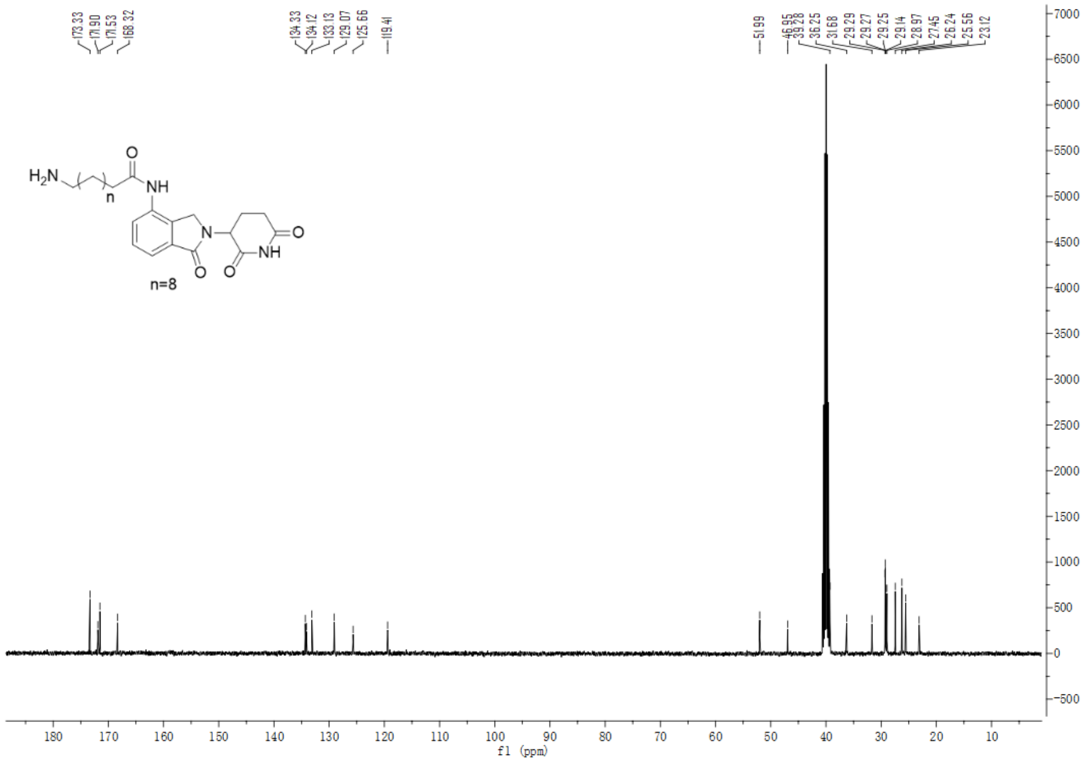
173.33
171.90
171.53
168.32
134.33
134.12
133.13
129.07
125.66
119.41
51.99
46.95
36.25
31.68
29.29
29.27
29.25
29.14
28.97
27.45
26.24
25.56
23.12
H2N
NH
N
NH
O
n
n=8
f1 (ppm)

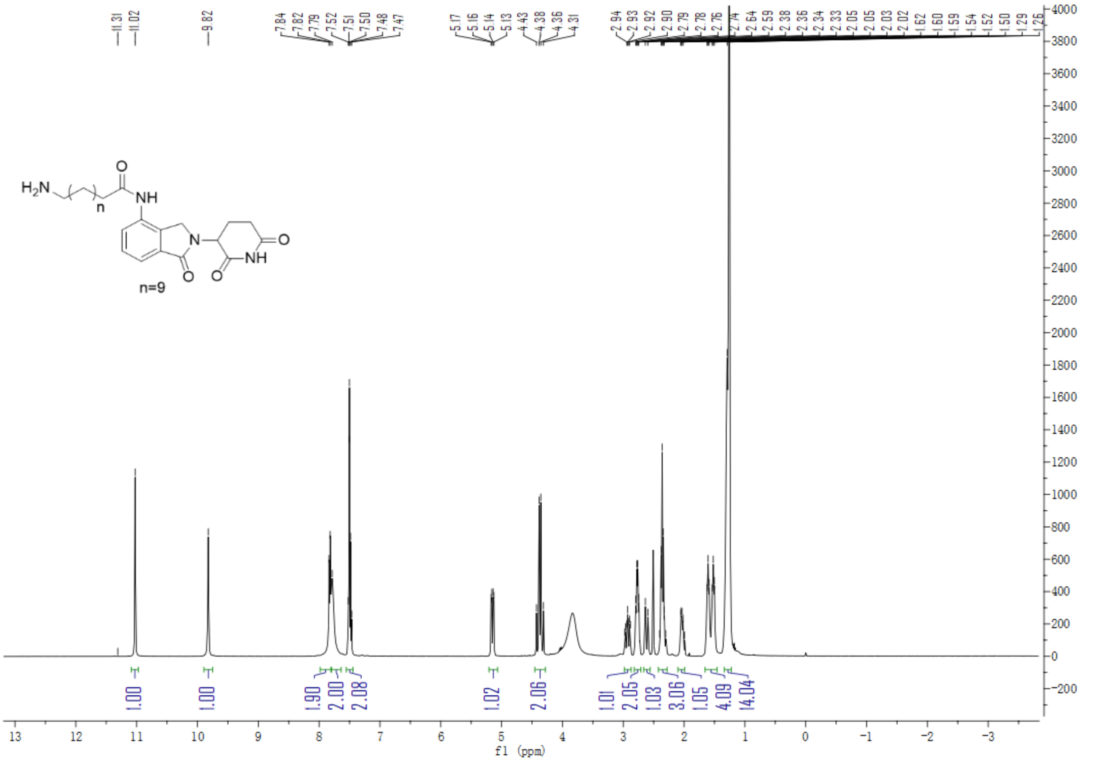
H2N
NH
N
NH
n
n=9
1.00
1.00
1.90
2.00
2.08
1.02
2.06
1.01
2.05
1.03
3.06
1.05
4.09
14.04
f1 (ppm)

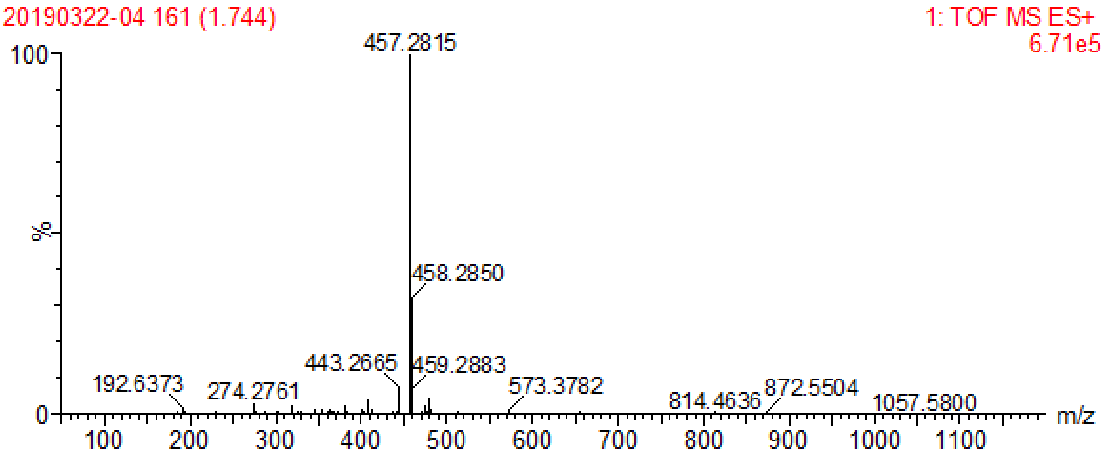
20190322-04 161 (1.744)
1: TOF MS ES+
6.71e5
457.2815
458.2850
443.2665
459.2883
192.6373
274.2761
573.3782
814.4636
872.5504
1057.5800
%
m/z

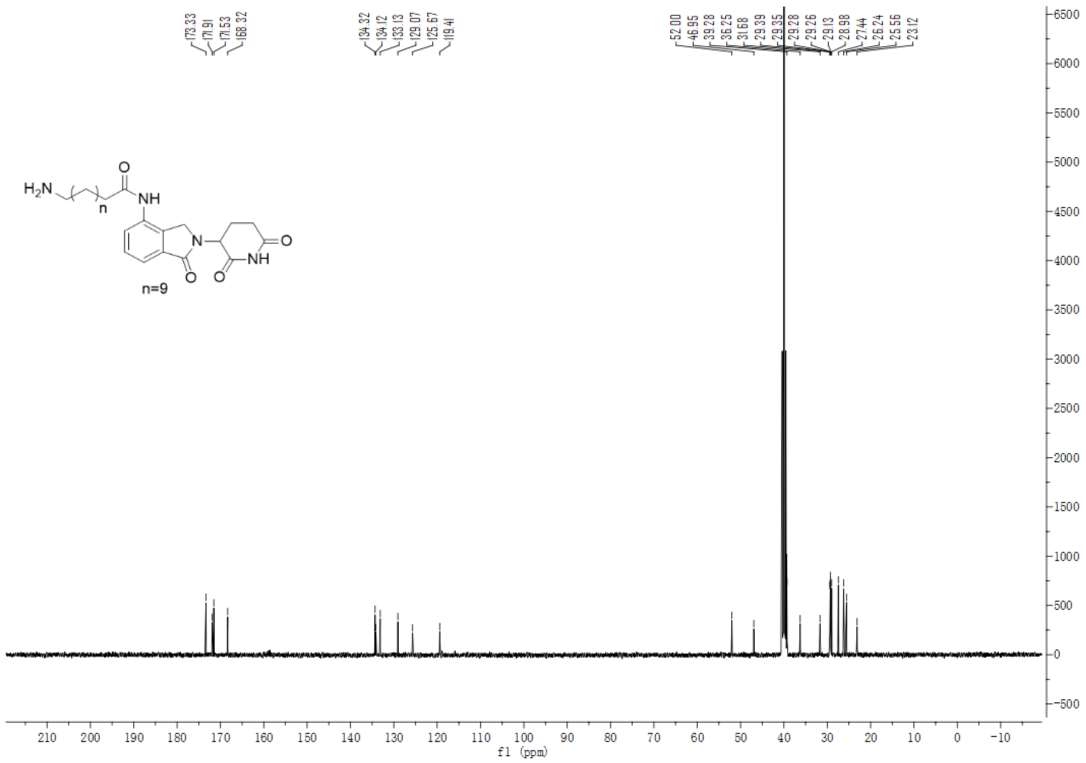

173.33
171.91
171.53
168.32
134.32
134.12
133.13
129.07
125.67
119.41
52.00
46.95
39.28
36.25
31.68
29.39
29.35
29.28
29.26
29.13
28.98
27.44
26.24
25.56
23.12
H_2N
NH
O
N
NH
n
n=9
f1 (ppm)

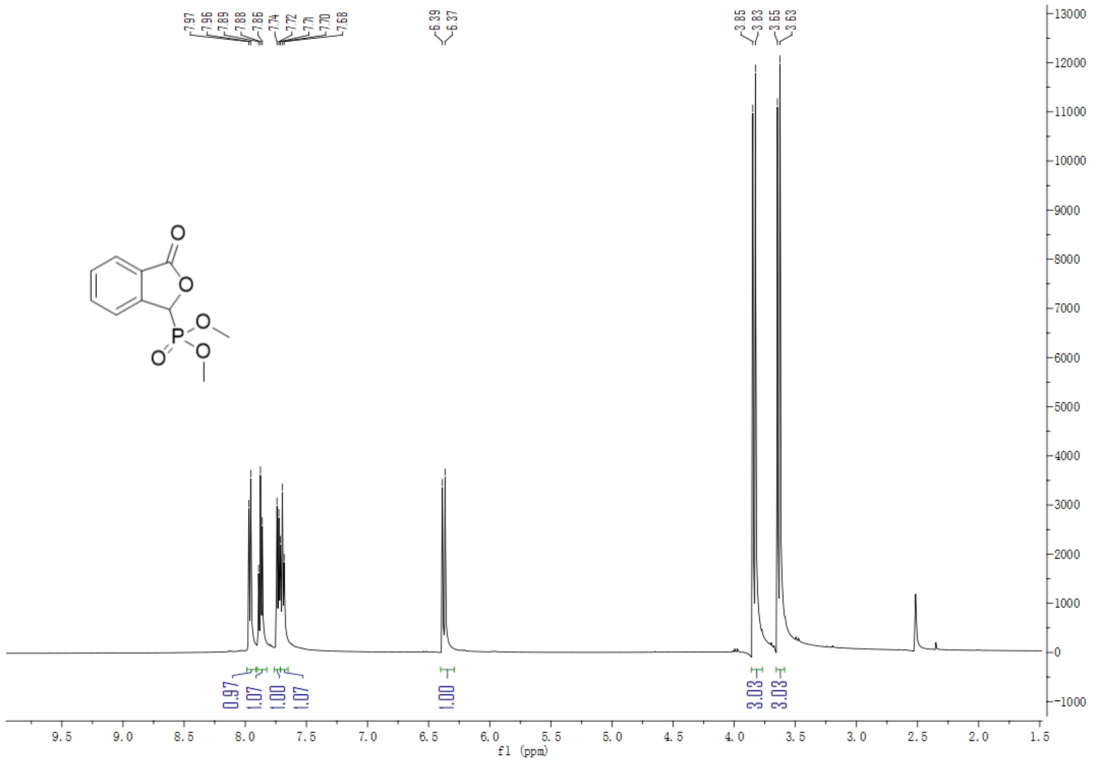
7.97
7.96
7.89
7.88
7.86
7.74
7.72
7.71
7.70
7.68
6.39
6.37
3.85
3.83
3.65
3.63
0.97
1.07
1.00
1.07
1.00
3.03
3.03
f1 (ppm)

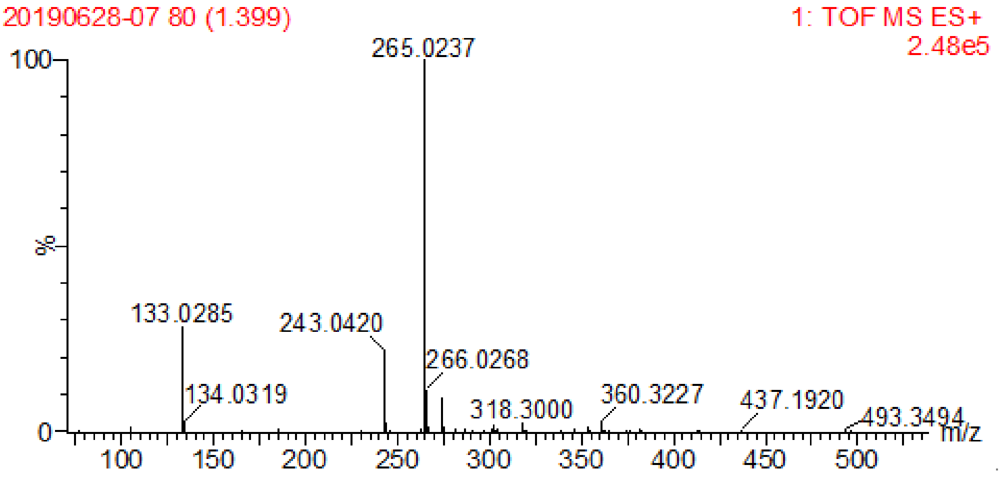
20190628-07 80 (1.399)
1: TOF MS ES+
2.48e5
265.0237
133.0285
243.0420
266.0268
134.0319
318.3000
360.3227
437.1920
493.3494
%
m/z

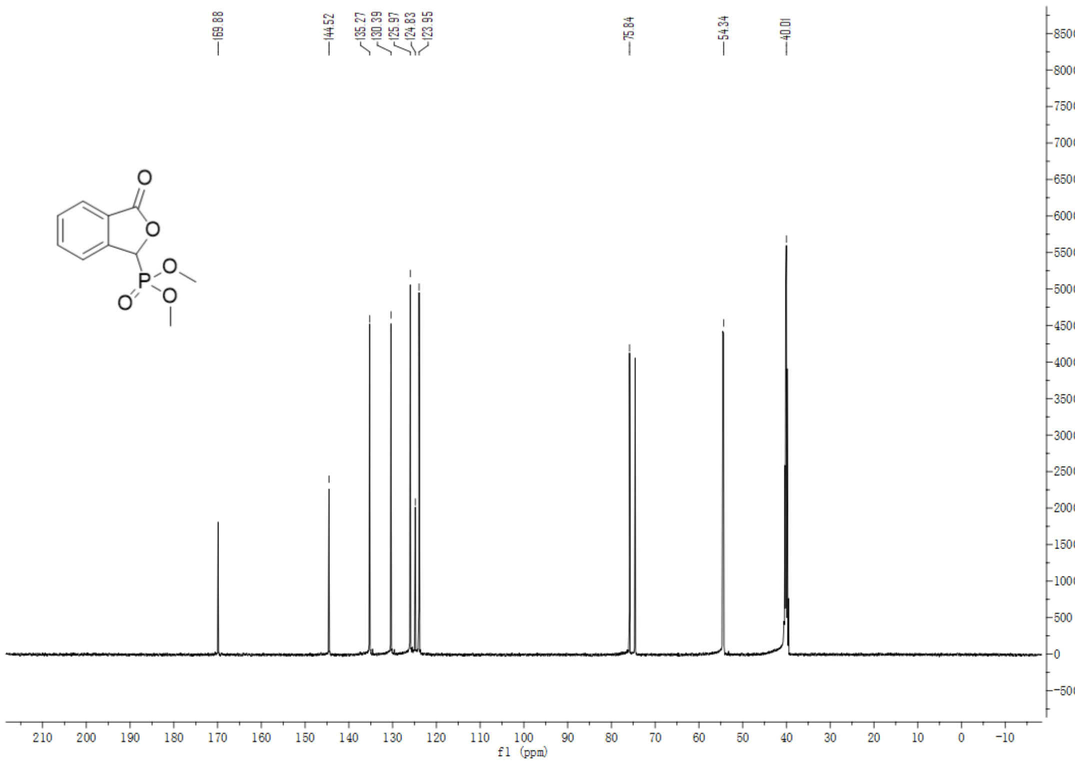
169.88
144.52
135.27
130.39
125.97
124.83
123.95
75.84
54.34
40.01
f1 (ppm)

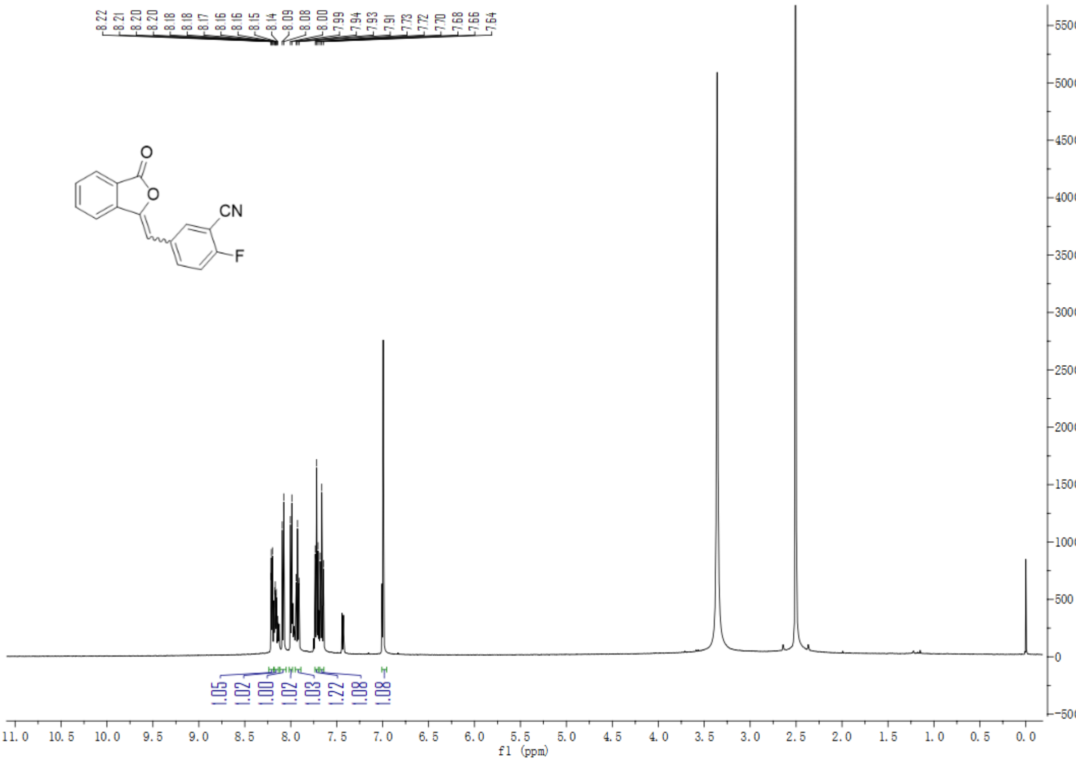
8.22 8.21 8.20 8.20 8.18 8.18 8.17 8.16 8.16 8.15 8.14 8.09 8.08 8.00 7.99 7.94 7.93 7.91 7.73 7.72 7.70 7.68 7.66 7.64
O
O
CN
F
1.05 1.02 1.00 1.02 1.03 1.22 1.08 1.08
11.0 10.5 10.0 9.5 9.0 8.5 8.0 7.5 7.0 6.5 6.0 5.5 5.0 4.5 4.0 3.5 3.0 2.5 2.0 1.5 1.0 0.5 0.0
f1 (ppm)
5500 5000 4500 4000 3500 3000 2500 2000 1500 1000 500 0 -500

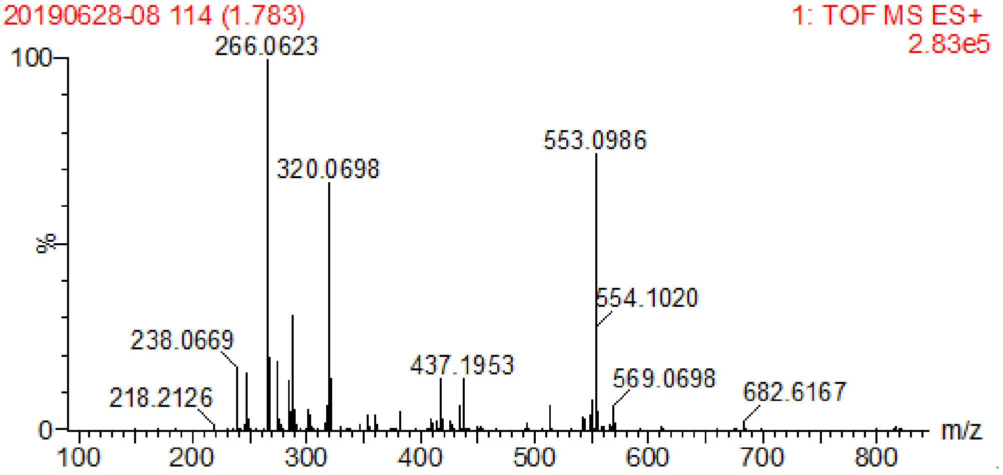
20190628-08 114 (1.783)
1: TOF MS ES+
2.83e5
266.0623
553.0986
320.0698
554.1020
238.0669
437.1953
569.0698
218.2126
682.6167
100
%
0
100 200 300 400 500 600 700 800
m/z

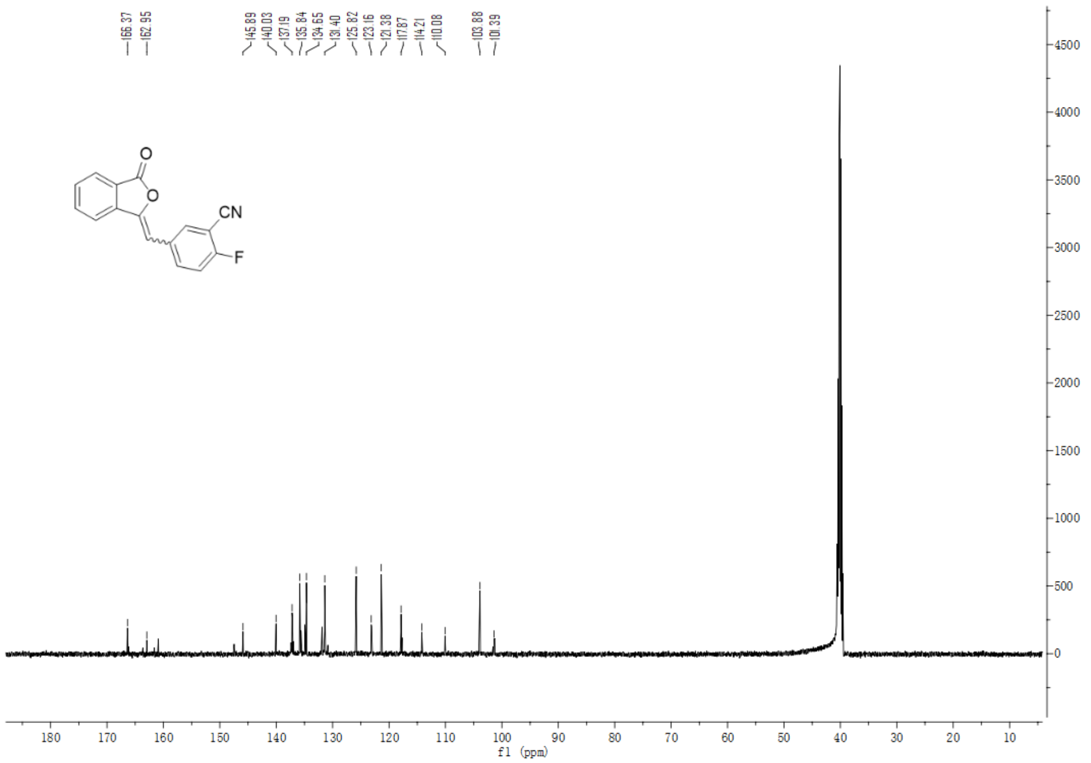

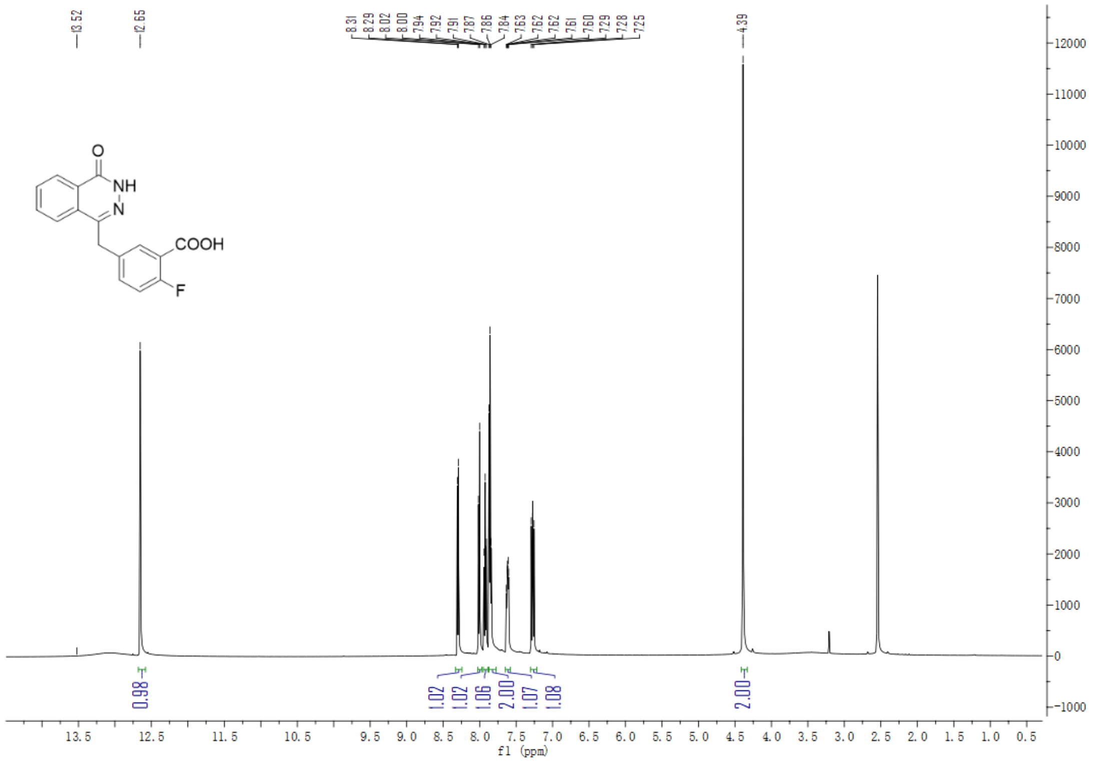

13.52
12.65
8.31
8.29
8.02
8.00
7.94
7.92
7.91
7.87
7.86
7.84
7.63
7.62
7.62
7.61
7.60
7.29
7.28
7.25
4.39
NH
N
COOH
F
0.98
1.02
1.02
1.06
2.00
1.07
1.08
2.00
f1 (ppm)

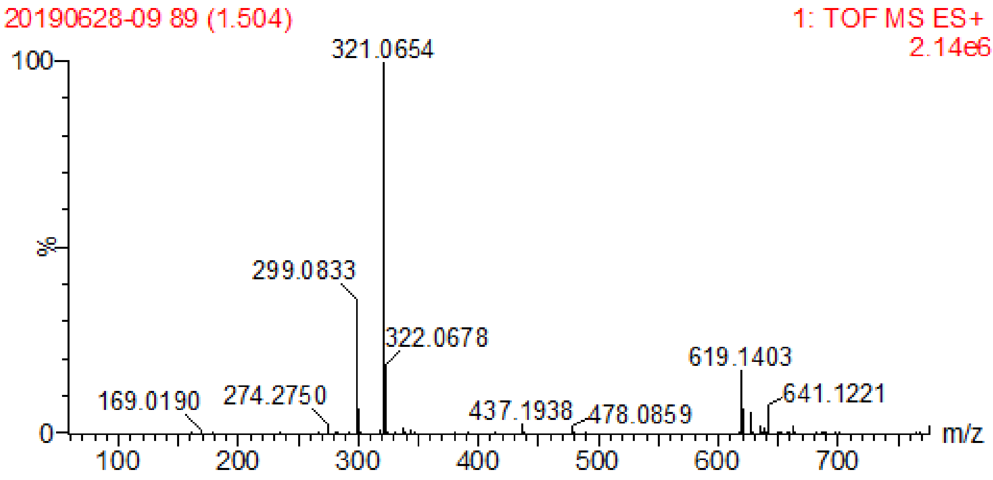

20190628-09 89 (1.504)
1: TOF MS ES+
2.14e6
321.0654
299.0833
322.0678
619.1403
641.1221
169.0190
274.2750
437.1938
478.0859
%
m/z

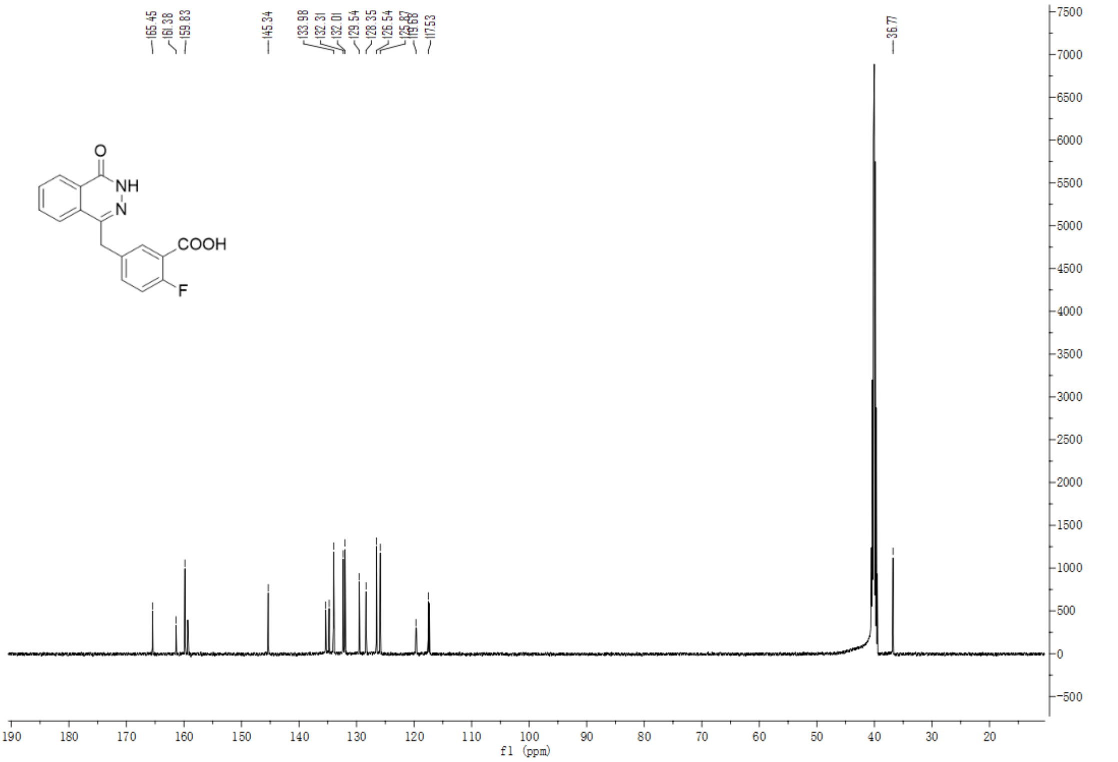
165.45
161.38
159.83
145.34
133.98
132.31
132.01
129.54
128.35
126.54
125.87
119.68
117.53
36.77
O
NH
N
COOH
F
7500
7000
6500
6000
5500
5000
4500
4000
3500
3000
2500
2000
1500
1000
500
0
-500
190 180 170 160 150 140 130 120 110 100 90 80 70 60 50 40 30 20
f1 (ppm)

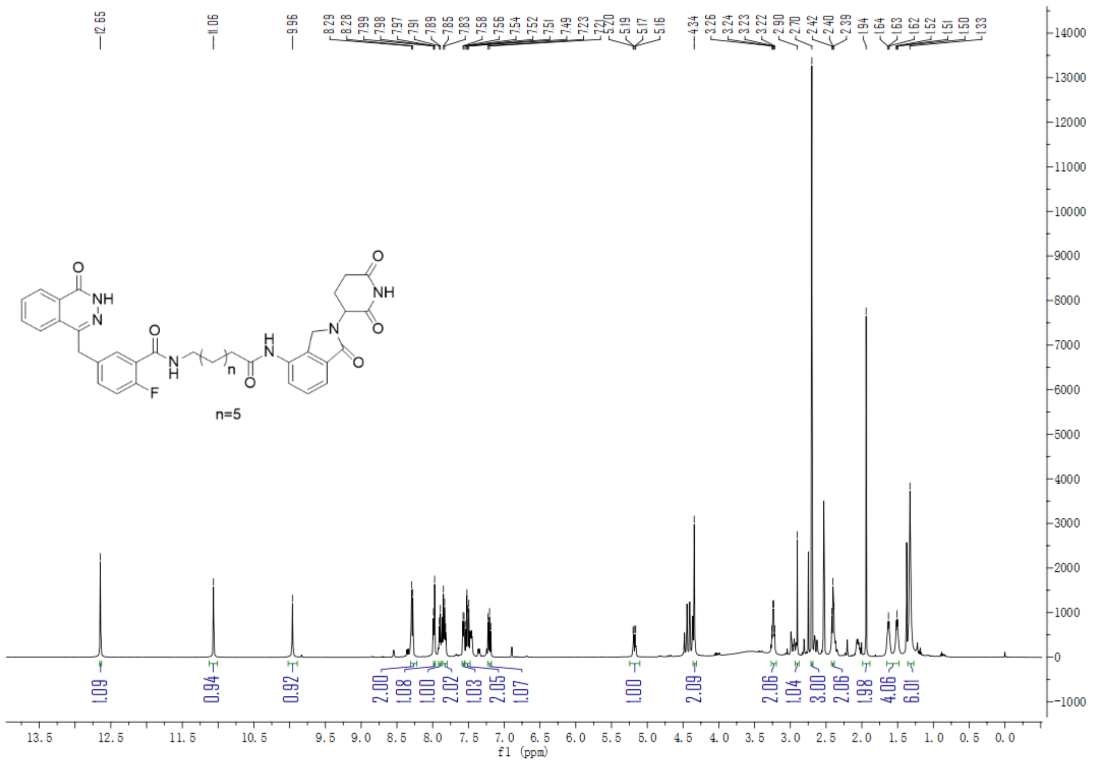
n=5
f1 (ppm)

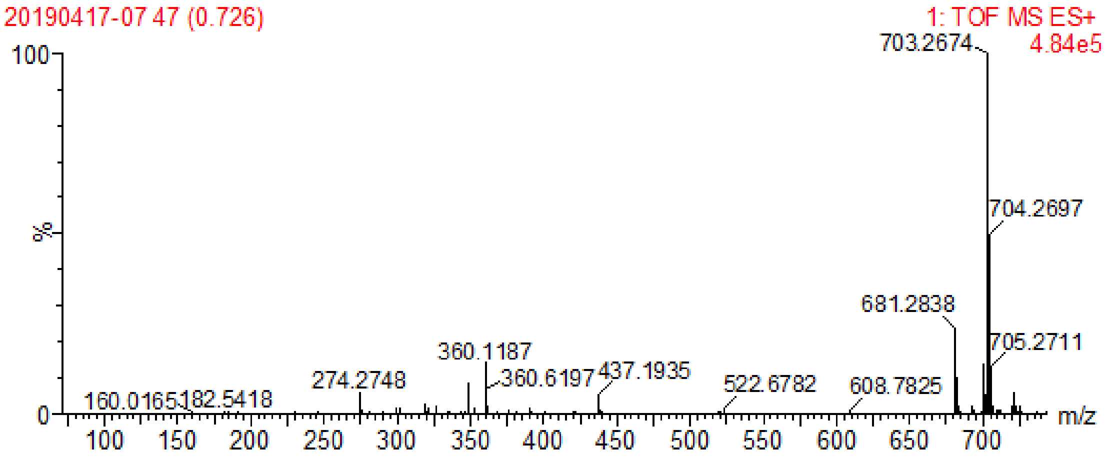
20190417-07 47 (0.726)
1: TOF MS ES+
4.84e5
703.2674
704.2697
681.2838
705.2711
360.1187
360.6197
437.1935
274.2748
522.6782
608.7825
160.0165
182.5418
m/z
%

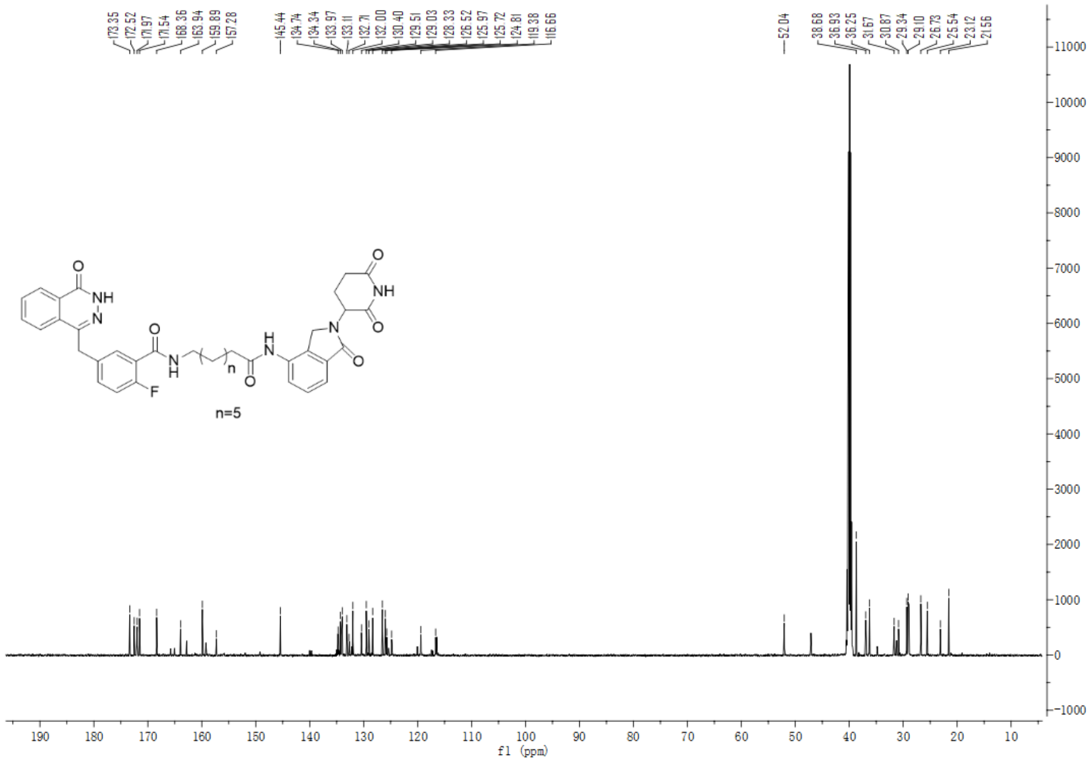
173.35
172.52
171.97
171.54
168.36
163.94
159.89
157.28
145.44
134.74
134.34
133.97
133.11
132.71
132.00
130.40
129.51
129.03
128.33
126.52
125.97
125.72
124.81
119.38
116.66
52.04
38.68
36.93
36.25
31.67
30.87
29.34
29.10
26.73
25.54
23.12
21.56
O
NH
N
O
N
H
F
n
O
H
N
N
O
NH
O
n=5
f1 (ppm)

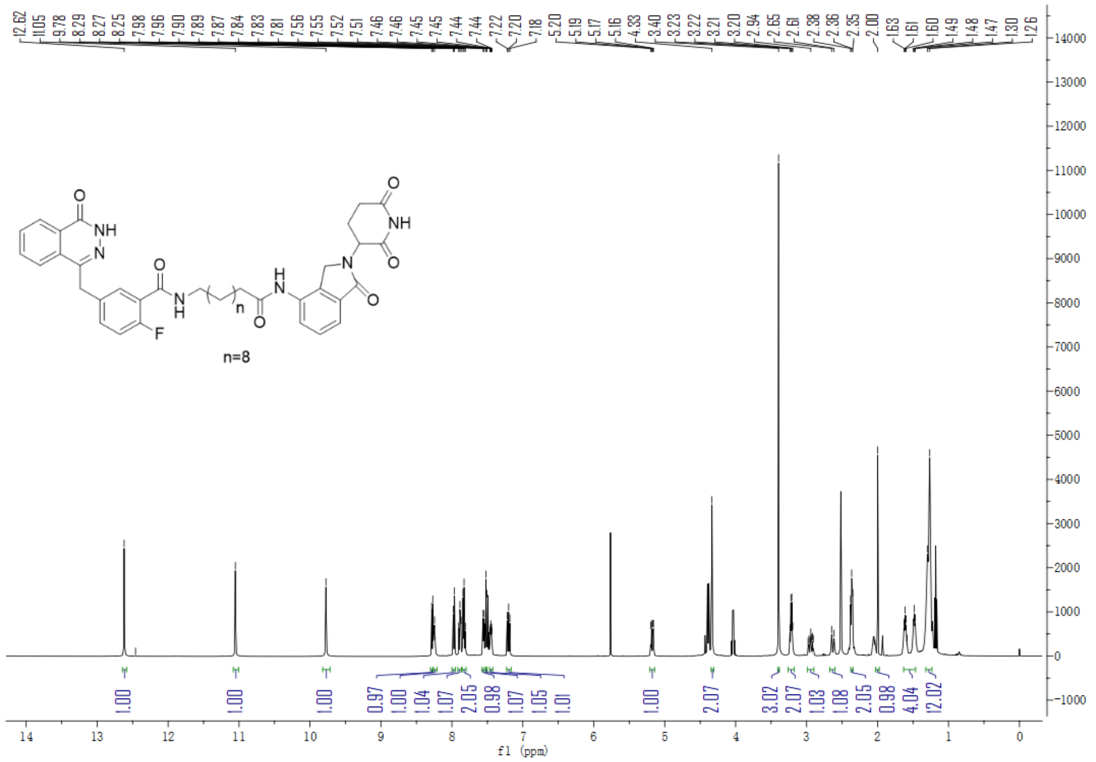
n=8
f1 (ppm)

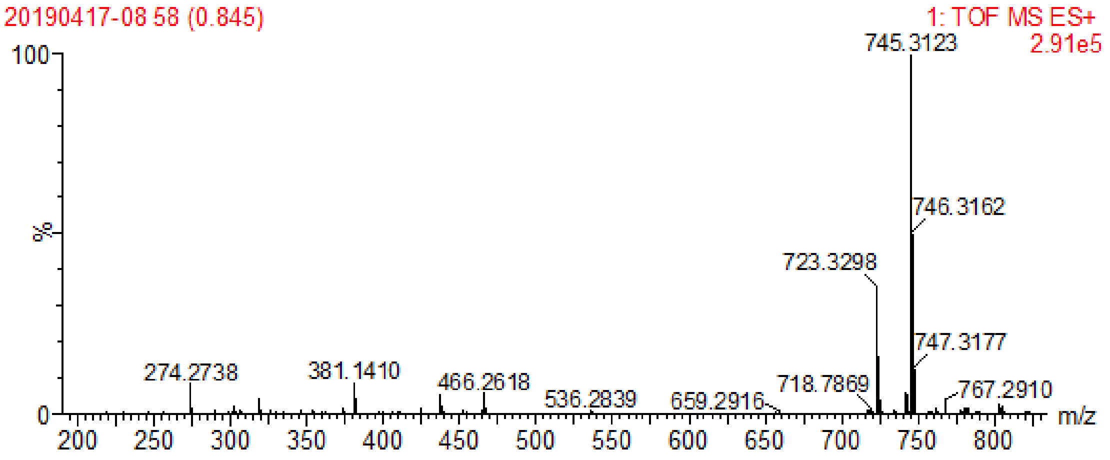
20190417-08 58 (0.845)
1: TOF MS ES+
2.91e5
745.3123
746.3162
723.3298
747.3177
718.7869
767.2910
274.2738
381.1410
466.2618
536.2839
659.2916
m/z

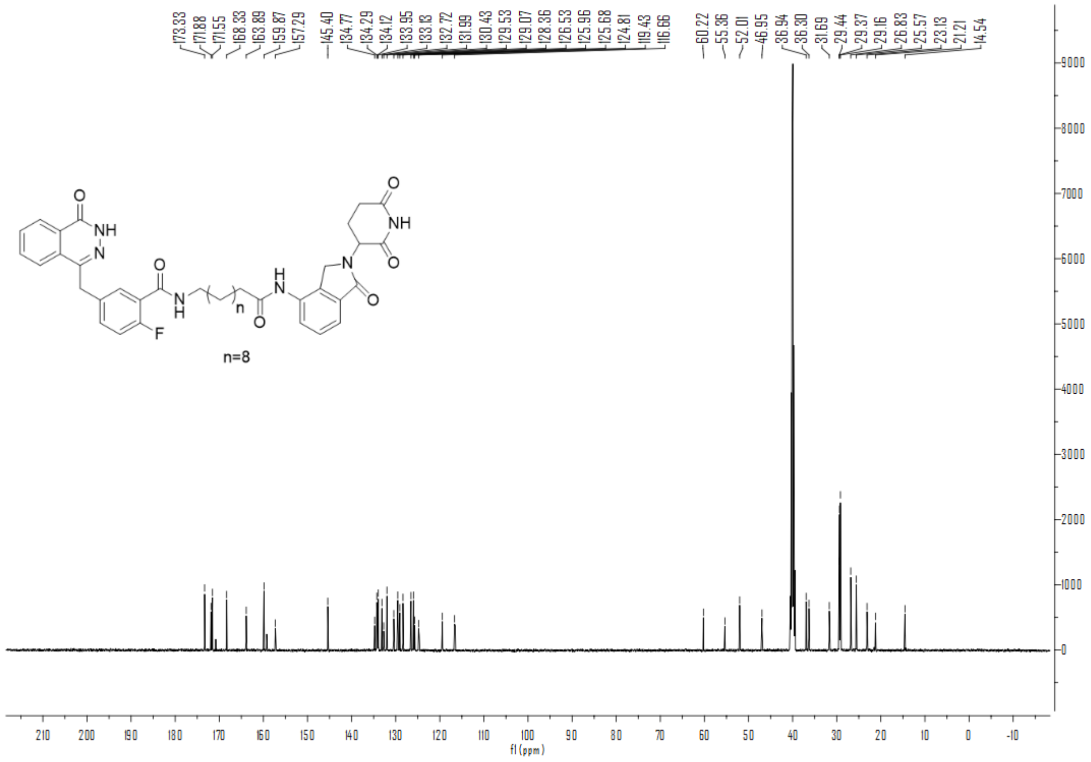

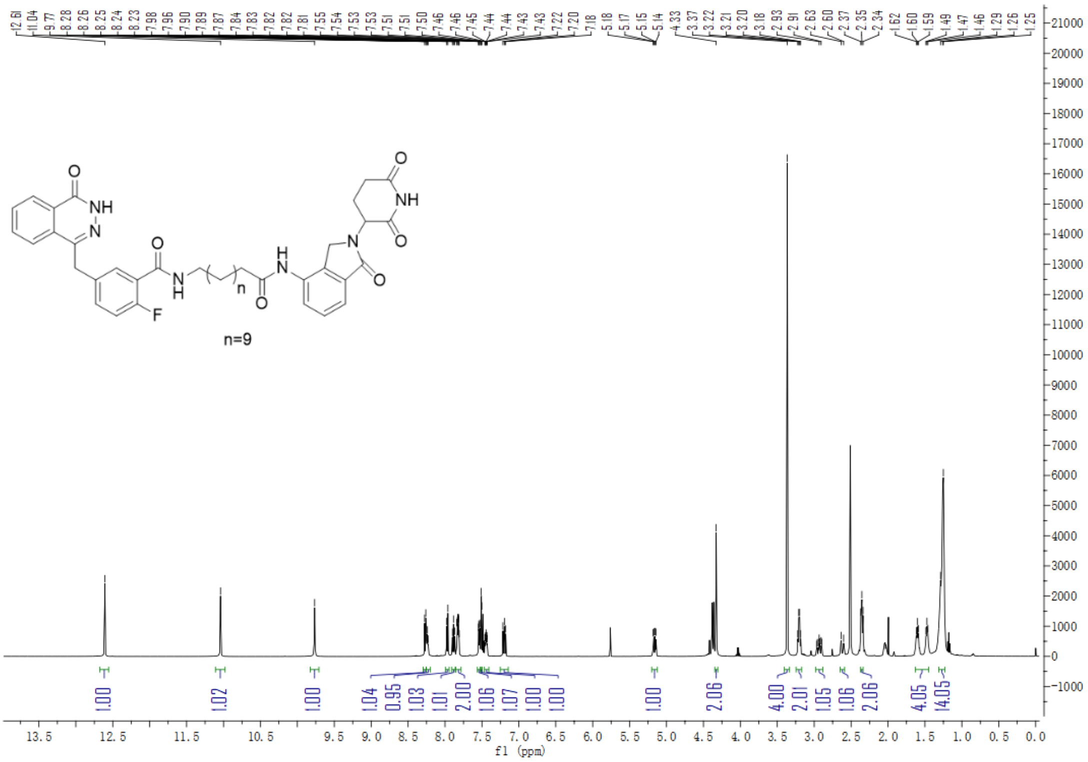
n=9
f1 (ppm)

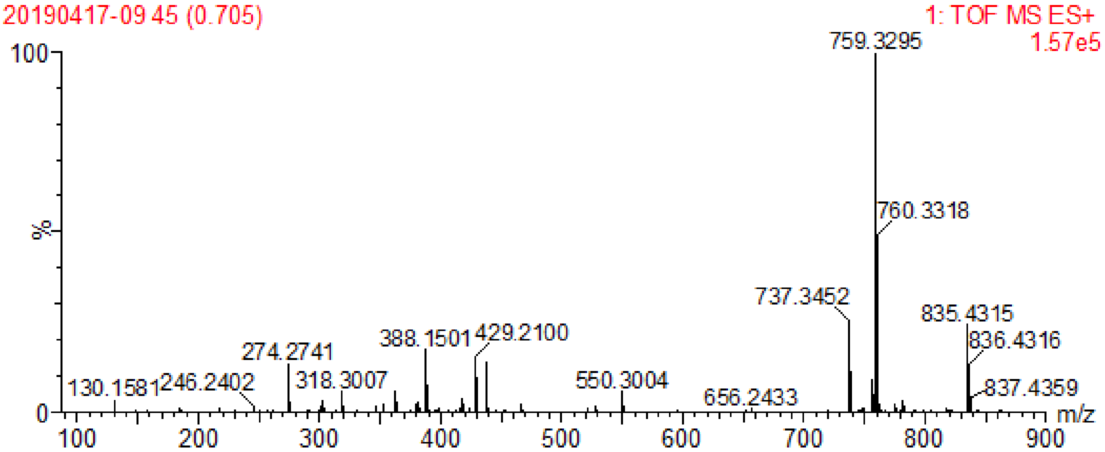
20190417-09 45 (0.705)
1: TOF MS ES+
1.57e5
759.3295
760.3318
737.3452
835.4315
836.4316
837.4359
429.2100
388.1501
274.2741
318.3007
130.1581
246.2402
550.3004
656.2433
%
m/z

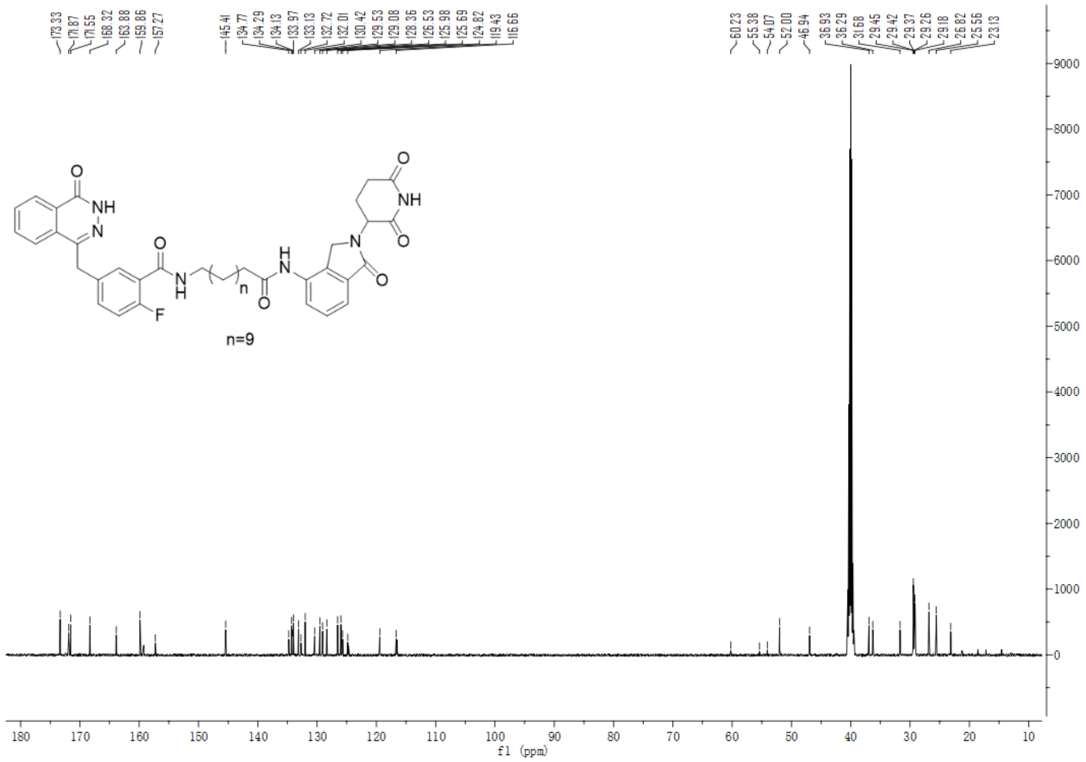

Supplement: Supplemental Material [file IENZ_A_1804382_SM9389.pdf]
